# Cost-effectiveness analysis of noninvasive tests to identify advanced fibrosis in non-alcoholic fatty liver disease

**DOI:** 10.1097/HC9.0000000000000191

**Published:** 2023-06-22

**Authors:** Lina Gruneau, Stergios Kechagias, Per Sandström, Mattias Ekstedt, Martin Henriksson

**Affiliations:** 1 Center for Medical Technology Assessment, Department of Health, Medicine and Caring Sciences, Linköping University, Sweden; 2 Division of Diagnostics and Specialist Medicine, Department of Health, and Caring Sciences, Linköping University, Sweden; 3 Division of Surgery, Department of Biomedical and Clinical Sciences, Orthopedics, and Oncology, Linköping University, Sweden; 4 Center for Medical Image Science and Visualization, Linköping University, Linköping, Sweden

## Abstract

Advanced fibrosis is associated with end-stage liver disease (ESLD) and mortality in NAFLD. As treatments specifically targeted at NAFLD are lacking, patient management focuses on surveillance for early detection of complications related to end-stage liver disease. Although current and emerging diagnostic tools for the detection of advanced fibrosis are crucial for surveillance, their added value is unclear. The aim of this study was to evaluate the costs and health outcomes of noninvasive tests in patient management strategies for diagnosing advanced fibrosis in NAFLD patients.

**Method:**

A decision analytical model was developed to evaluate 13 patient management strategies, including a no-testing strategy and 12 diagnostic algorithms with noninvasive tests (fibrosis 4- score, enhanced liver fibrosis, vibration controlled transient elastography), and liver biopsy. Model inputs were synthesized from the literature and Swedish registries. Lifetime health care costs, life years, quality-adjusted life years, clinical outcomes, and incremental cost-effectiveness ratios were calculated for a cohort of 55-year-old patients diagnosed with NAFLD.

**Result:**

The cost per quality-adjusted life year was above €50 000 for all diagnostic algorithms compared to no-testing. The cost per quality-adjusted life year of the most promising diagnostic algorithm (fibrosis 4- score, enhanced liver fibrosis, vibration controlled transient elastography, and liver biopsy) was ∼ €181 000 compared with no testing. Sensitivity analysis indicated that if treatment slowed down disease progression, the value of testing increased.

**Conclusion:**

The result questions the overall value of comprehensive diagnostic testing in a broad NAFLD population in current routine clinical care. The role of noninvasive tests may change if evidence-based treatments to slow down disease progression emerge.

## Graphical abstract

**Figure:**
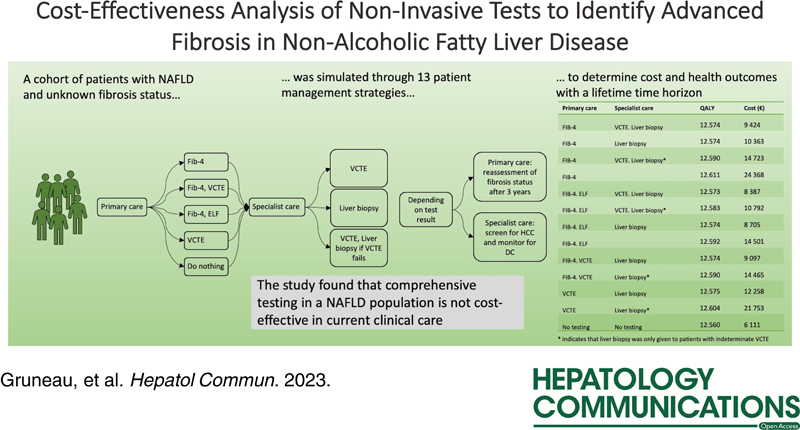

## INTRODUCTION

NAFLD is prevalent in 25% of the Western population, and disease progression is generally slow.^1,2^ Yet, for patients progressing to NASH, the risk of developing liver fibrosis increases.^3,4^ Advanced fibrosis is associated with an increased risk of severe liver disease, HCC, and other comorbidities such as cardiovascular disease, and mortality.^3,5,6^

Although some pharmacological treatments are in development,^7^ there are yet no treatment options targeted specifically at NASH/NAFLD.^8,9^ Hence, current care relies on early detection of NAFLD and fibrosis staging, enabling screening for HCC and esophageal varices, where early identification has a significant impact on survival.^10,11^ Diagnostic tools for advanced fibrosis are, therefore, a key component in the management of NAFLD. Liver biopsy has long been considered the gold standard,^12,13^ but it is subjected to sampling errors as well as being costly and invasive.^14,15^ Noninvasive tests (NITs) are rapidly emerging, and their role in long-term NAFLD management is likely to be more pronounced in the future. These are not used in isolation or at a particular point in time; rather, diagnostic algorithms encompass testing and surveillance both in primary and specialist care.^13,16^

Previous studies have assessed the cost-effectiveness of diagnostic algorithms in a screening setting^17,18^ as well as in a clinical care setting in patients with confirmed NAFLD.^19,20^ However, these studies did not evaluate noninvasive diagnostic algorithms performed in sequence in primary and specialist care, as proposed by guidelines.^13,21,22^ Furthermore, guidelines propose that patients with NAFLD in primary care who have not been initially diagnosed with advanced fibrosis but with underlying NAFLD should be retested for advanced fibrosis after a set time to identify progressed patients.^13^ This aspect is lacking in previous studies. Therefore, the cost-effectiveness of diagnostic algorithms during the complete health care management trajectory for patients with NAFLD is not known. The overall aim of this study was to evaluate the costs and health outcomes of NITs for the assessment of advanced fibrosis in patients with NAFLD. Our study adds to the existing literature by evaluating a broader range of diagnostic algorithms and incorporating a more thorough health care management trajectory for patients identified with NAFLD.

## METHODS

### Overview of economic evaluation methods

To appropriately evaluate the health care costs and ultimate health outcomes associated with different diagnostic algorithms, several candidates must be evaluated and compared in a cost-effectiveness analysis.^23^ Such an evaluation requires a long-term time horizon and the synthesizing of evidence from various sources in a decision analytical modelling framework.^23,24^

Health outcomes were measured as quality-adjusted life years (QALY), combining the length of life and quality of life. Health care costs included diagnostics, primary care, outpatient care, and hospitalizations. Costs and QALYs were estimated for a lifetime time horizon and were discounted by 3% per annum.^25^ Incremental cost-effectiveness ratios were calculated after excluding dominated and extendedly dominated alternatives.^24^ The net health benefit (NHB) was calculated using a cost-effectiveness threshold of €50 000 per QALY. Additional outcomes were life years gained, number of decompensated cirrhosis (DC) cases, number of detected HCC cases (by surveillance program or opportunistically), number of liver transplants, frequency of treatments, and 10-year mortality.

### Patients and diagnostic strategies

**FIGURE 1:**
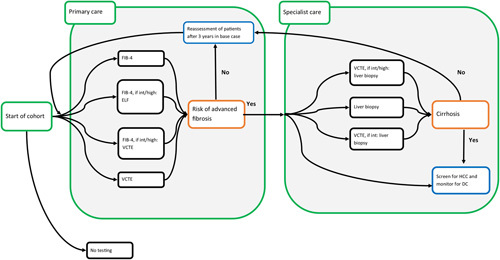
Flow chart of evaluated diagnostic algorithms. Abbreviations: FIB-4, Fibrosis 4 index; ELF, enhanced liver fibrosis; VCTE, vibration controlled transient elastography; Int, indeterminate.

The population of interest was patients clinically diagnosed with NAFLD of unknown fibrosis stage and negative assessments for alcohol abuse, hepatitis C, hepatitis B, and DILI. For these patients, 13 patient management strategies were evaluated, 12 diagnostic algorithms, and a no-testing strategy (Figure 1). The 12 diagnostic algorithms combined Fibrosis 4 index (FIB-4), Enhanced Liver fibrosis test (ELF), Vibration Controlled Transient Elastography (VCTE), and biopsy. Noninvasive tests could be performed in primary or specialist care (Table 1). In the no-testing strategy, no testing was conducted for diagnosing fibrosis stage.

**TABLE 1:** Summary of patient management strategies

Strategy	Primary care	Specialist care	Description
1	FIB-4	VCTE, Liver biopsy	Patients with indeterminate VCTE or with VCTE value above the threshold undergo biopsy.
2	FIB-4	Liver biopsy	Patients with FIB-4 scores above the threshold undergo biopsy.
3	FIB-4	VCTE, Liver biopsy	Patients with indeterminate VCTE undergo biopsy.
4	FIB-4	—	Surveillance for all patients with FIB-4 scores above the threshold.
5	FIB-4, ELF	VCTE, Liver biopsy	Patients with indeterminate VCTE or with VCTE value above the threshold undergo biopsy.
6	FIB-4, ELF	VCTE, Liver biopsy	Patients with indeterminate VCTE undergo biopsy.
7	FIB-4, ELF	Liver biopsy	Surveillance for all patients with ELF scores above the threshold.
8	FIB-4, ELF	—	Surveillance for all patients with ELF scores above the threshold.
9	FIB-4, VCTE	Liver biopsy	Patients with indeterminate VCTE or with VCTE values above the threshold undergo biopsy.
10	FIB-4, VCTE	Liver biopsy	Patients with indeterminate VCTE undergo biopsy.
11	VCTE	Liver biopsy	Patients with indeterminate VCTE or with VCTE values above the threshold undergo biopsy.
12	VCTE	Liver biopsy	Patients with indeterminate VCTE undergo biopsy.
13	No testing	No testing	—

Abbreviations: ELF, enhanced liver fibrosis; FIB-4, fibrosis 4 index; VCTE, vibration-controlled transient elastography.

### Disease progression and impact of diagnostics

**FIGURE 2:**
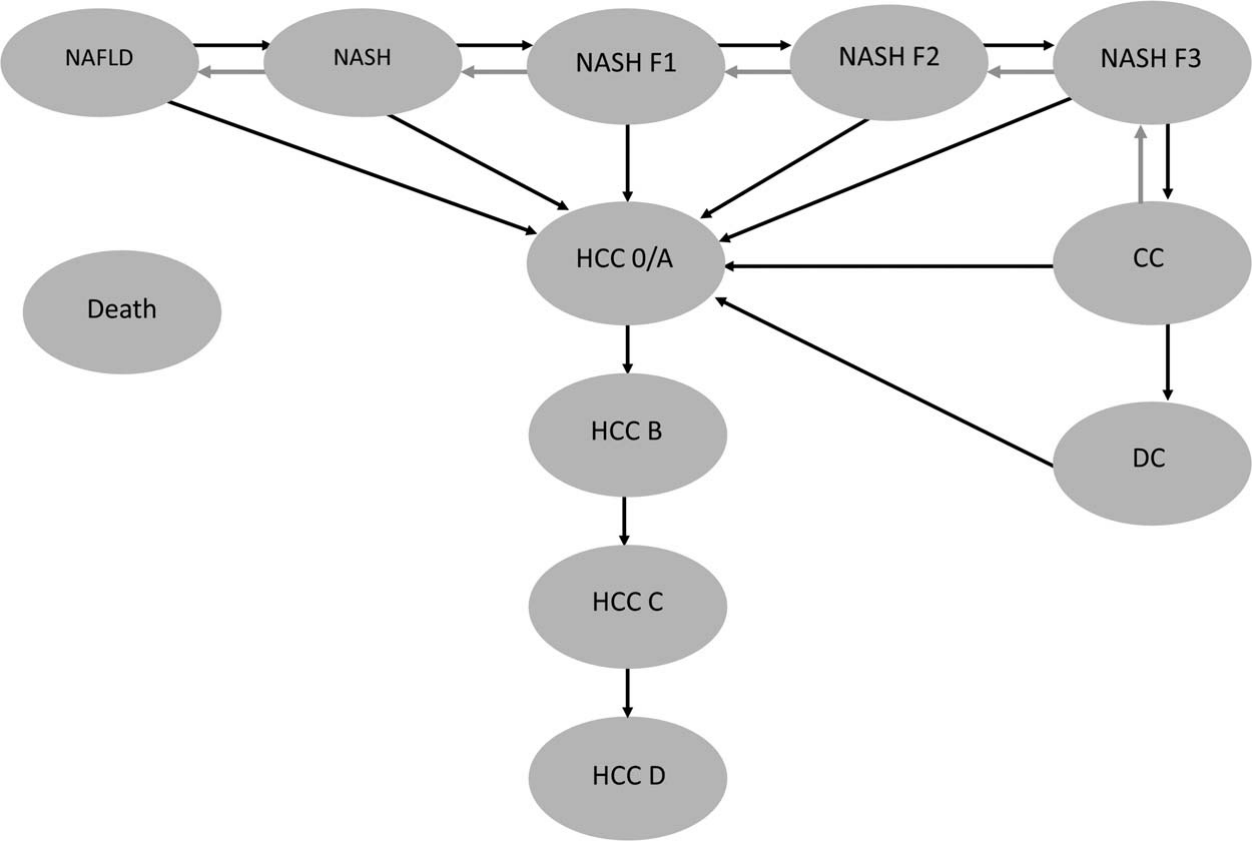
Markov state transition model. Abbreviations: F, fibrosis; CC, compensated cirrhosis; DC, decompensated cirrhosis.

To estimate long-term costs and health outcomes of the diagnostic algorithms the natural history of NAFLD was modelled using a Markov structure; a series of mutually exclusive health states, represented by ovals in Figure 2. An individual could reside in one state in a given time period (a year in this analysis),^24^ and transition between states from one time period to another, as indicated by the arrows in Figure 2. Each health state or transition between health states were associated with a cost and a QALY-weight enabling the estimation of accumulated long-term costs and QALYs as a cohort of NAFLD patients progressed through the model with each patient management strategy. The model structure was based on previous NAFLD disease progression models and was considered a good compromise between parsimony and complexity.^26^

In the modelled disease progression, patients could transition between NAFLD, NASH, NASH Fibrosis 1 (NASH F1), NASH fibrosis 2 (NASH F2), NASH fibrosis 3 (NASH F3), compensated cirrhosis (CC), decompensated cirrhosis (DC), and HCC with and without cirrhosis (Figure 2). HCC was modelled according to Barcelona Clinic Liver Cancer in stages 0/A, B, C, D.^27^ Patients were allowed to regress in their fibrosis and transition back to a less advanced fibrosis stage as part of natural disease course, see Supplemental table S1, http://links.lww.com/HC9/A332 for detailed transitions. A proportion of patients with DC were eligible for liver transplantation. Patients with HCC were treated with liver transplantation, other curative treatment (ablation or resection), or non-curative treatment (transcatheter arterial chemoembolization (TACE), sorafenib or other best supportive care) based on the Barcelona Clinic Liver Cancer stage. In every cycle of the model individuals faced a mortality risk subject to disease status, treatment regimen, and age.

Disease stage prevalence data^18^ determined the proportion of patients in the NAFLD cohort starting in different states of the model (Table 2). A cohort of hypothetical patients was subjected to all 13 patient management strategies and underwent testing at the start of the analysis. Patients identified with advanced fibrosis (false positive and true positive) were retained in specialist care. In specialist care patients underwent a surveillance program for HCC detection and received DC prevention according to guidelines.^13,21,22^ For patients classified as non-advanced (false negative and true negative) disease status was reassessed in primary care every three years in the base case scenario. Patients in the no-testing strategy were not tested for fibrosis status.

**TABLE 2:** Selected input parameters

Parameter	Value	Source
Prevalence		
NAFLD	0.215	^18^
NASH	0.145	^18^
F1	0.305	^18^
F2	0.174	^18^
F3	0.134	^18^
CC	0.027	^18^
Test characteristics		
FIB-4		
NAFLD-F3 < 65y (sensitivity/specificity)	0.690/0.700	^28^
NAFLD-F3 > 65y (sensitivity/specificity)	0.770/0.700	^29^
CC (sensitivity)	0.870	Assumption^28^
ELF		
NAFLD-F3 (sensitivity/specificity)	0.730/0.800	^30^
CC (sensitivity)	0.900	Assumption^31^
VCTE		
NAFLD-F3 (sensitivity/specificity)	0.770/0.78	^28^
CC (sensitivity)	0.900	Assumption^28^
Indeterminate result	0.158	^32^
Liver biopsy		
NAFLD-CC (sensitivity/specificity)	1/1	Assumption^17^
Death caused by liver biopsy	0.0001	^33^
Cost of diagnostic tests (€)		
FIB-4	70	Local data^34^
ELF	99	^34,35^
VCTE (primary care)	221	^34,36^
VCTE (specialist care)	421	^34,36^
Liver biopsy	1 375	^34^

Abbreviations: CC, compensated cirrhosis; DC, decompensated cirrhosis; ELF, enhanced liver fibrosis; F, fibrosis; FIB-4, fibrosis 4 index; VCTE, vibration controlled transient elastography.

Due to the prominent symptoms, it was assumed that patients developing DC were immediately identified and treated accordingly in specialist care. In addition, patients developing HCC outside of the surveillance program in specialist care faced an annual probability of being diagnosed opportunistically that increased with the Barcelona Clinic Liver Cancer stage. Additional details of the modelled disease progression and the impact of patient management strategies can be found in the supplemental material, http://links.lww.com/HC9/A332.

### Data sources and model inputs

#### Prevalence and disease progression

Disease progression probabilities were derived from the literature (see Supplemental tables S1, and S2, http://links.lww.com/HC9/A332 for details) and relied on commonly used sources.^26^ The proportions of HCC patients being transplanted, receiving other curative and non-curative treatment, by HCC stage, were derived from the Swedish Registry SweLiv.^37^ The same registry was used to estimate the survival prognosis associated with each treatment. Other mortality rates in the model were derived from national statistics population data^38^ and HRs representing additional disease stage risk applied for patients with NAFLD, NASH, NASH fibrosis, and CC were retrieved from the literature.^39^ For patients with ESLD, mortality was conditional on disease stage only and not age. When lack of data required assumption, clinical experts were consulted.

#### Test characteristics

Test characteristics applied in the model are reported in Table 2. Cut-offs for the noninvasive diagnostic tests were based on Youden-index. FIB-4 relies on routine clinical and laboratory parameters,^31,40^ and sensitivity and specificity were derived from a meta-analysis study using liver biopsy as benchmark.^28^ Cut-off values of FIB-4 were 1.440 for patients < 65 yrs. and 2.000 for patients ≥ 65 yrs.^13^ ELF is a blood test used to assess markers for fibrosis.^41^ Sensitivity and specificity were derived from a meta-analysis of ELF, using liver biopsy as benchmark, and cut-off for detection of advanced fibrosis was 9.370.^30^ VCTE was assumed to be measured by FibroScan. A cut-off of 9.100 kPa was used in the study deriving sensitivity and specificity with biopsy as benchmark.^28^ Confirmatory liver biopsy was assumed to have 1/1 sensitivity/specificity. Due to the invasive nature of liver biopsy patients faced a (small) mortality risk associated with the procedure.^33^ Patients over 80 years of age were not eligible for liver biopsy and the preceding NIT-result were used to classify patients that did not undergo biopsy due to age. When VCTE were the preceding diagnostic test, patients with indeterminate results were assumed to be retested and with a second indeterminate result a negative result was assumed (true or false negative). Indeterminate results of VCTE were defined as unreliable results with fewer than 10 valid shots.^32^

#### Cost

Key cost parameters included cost of diagnostic tests (Table 2), and follow-up in primary- and specialist care (Supplemental table S3, http://links.lww.com/HC9/A332). The number of outpatient visits was derived from a study on health care cost of patients with NAFLD.^42^ Follow-up in specialist care included cost of screening every 6 months for HCC and management of DC. The costs of treatment for HCC, DC, and liver transplant were derived from various sources as detailed in Supplemental table S3, http://links.lww.com/HC9/A332. Costs in specialist care were derived using action codes and ICD-10 related costs relying on ICD-10 codes from previous literature.^43^ All costs were converted to euro (€) and 2019 prices.

#### Quality of life

There are a general lack of quality-of-life estimates for patients with NAFLD, especially for later stages of the disease. QALY-weights were based on previous studies and age adjusted using recent Swedish general population data (see Supplemental tables S4, and S5, http://links.lww.com/HC9/A332).

### Analysis

We simulated a hypothetical cohort of 10 000 55-year-old men and women. Outcomes were estimated as per patient means per strategy. The model was developed in R (version 4.2.1) and based on previous work on state cohort-transition models in R.^44^ The model and data analysis were conducted in accordance with guidelines for cost-effectiveness modelling.^45^ One-way sensitivity analyses were conducted to analyze how structural assumptions and single parameters impacted the results. To capture the uncertainty in all relevant parameters jointly, a probabilistic sensitivity analysis was conducted by sampling 10 000 values from the probability distributions of the model parameters that reflected the precision with which they were estimated^24^ (Supplemental tables S1 to S5, http://links.lww.com/HC9/A332). Model validity was assessed by comparing estimated disease incidence and mortality rates with reported registry data and previous economic evaluation models, see Supplemental tables S6 to S9, http://links.lww.com/HC9/A332. Clinical endpoints predicted from the model are extensively reported to facilitate comparison of model output with other sources.

### Ethics

The study used data from a previous study on treatment patterns in patients with HCC.^37^ The ethical approval for the study was granted by the ethical vetting board at Linköping University (Dnr 2017/29-31).

## RESULTS

### Base-case analysis

The main results are reported in Table 3. In terms of clinical endpoints, the predicted number of DC cases was highest with the no-testing strategy as patients miss out on preventative care of DC with this strategy. The estimated 10-year mortality rate with no testing was 1 365 of 10 000, a rate that was reduced to 1 329 of 10 000 with the clinically most effective diagnostic algorithm (for additional mortality data see Supplemental tables S6, S7, S10, and S11, http://links.lww.com/HC9/A332). Without testing patients were diagnosed with HCC at a later stage and fewer received treatment compared with the diagnostic algorithms. It should be noted that the number of liver transplants with this strategy was also driven by the higher incidence of DC.

**TABLE 3:** Clinical outcomes and cost-effectiveness outcomes

Patient management strategies			Clinical outcomes (cases per 10 000)								Cost-effectiveness outcomes (per patient mean)			
Strategy	Primary care	Specialist care	Number of DC	Number of HCC	Number of detected HCC (surv)	Number of detected HCC (not surv)	Number of LT	Number of OCT	Number of NCT	10-year mortality	QALY	Cost (€)	Life years	NHB
1	FIB-4	VCTE. Liver biopsy	447.739	668.568	105.835	78.944	29.731	59.584	109.504	1354.937	12.574	9 424	15.069	12.385
2	FIB-4	Liver biopsy	443.579	668.416	118.541	77.117	31.096	65.796	111.882	1354.573	12.574	10 363	15.070	12.367
3	FIB-4	VCTE. Liver biopsy^a^	427.495	667.975	233.228	54.526	45.247	131.951	122.896	1344.383	12.590	14 723	15.098	12.296
4	FIB-4		411.080	667.567	386.741	25.550	67.117	221.642	133.796	1328.738	12.611	24 368	15.135	12.124
5	FIB-4. ELF	VCTE. Liver biopsy	452.191	668.688	99.130	80.092	29.277	56.811	107.548	1355.627	12.573	8 387	15.068	12.405
6	FIB-4. ELF	VCTE. Liver biopsy^a^	437.340	668.250	171.976	65.758	37.237	97.353	116.818	1349.278	12.583	10 792	15.084	12.367
7	FIB-4. ELF	Liver biopsy	448.416	668.589	105.014	79.093	29.688	59.267	109.233	1354.988	12.574	8 705	15.069	12.400
8	FIB-4. ELF		426.381	667.957	242.017	52.831	46.355	137.085	123.663	1343.309	12.592	14 501	15.100	12.302
9	FIB-4. VCTE	Liver biopsy	447.739	668.568	105.835	78.944	29.731	59.584	109.504	1354.937	12.574	9 097	15.069	12.392
10	FIB-4. VCTE	Liver biopsy^a^	427.495	667.975	233.228	54.526	45.247	131.951	122.896	1344.383	12.590	14 465	15.098	12.301
11	VCTE	Liver biopsy	441.660	668.384	117.346	77.126	30.713	64.727	112.332	1354.199	12.575	12 258	15.070	12.329
12	VCTE	Liver biopsy^a^	414.192	667.657	338.294	34.953	59.819	192.618	131.377	1334.297	12.604	21 753	15.122	12.169
13	No testing	No testing	516.696	701.399	40.988	73.960	28.155	38.837	82.634	1364.660	12.560	6 111	15.050	12.438

*Notes:* NHB calculated with a threshold of €50 000.

a Indicates that liver biopsy was only given to patients with indeterminate VCTE.

Abbreviations: DC, decompensated cirrhosis; ELF, enhanced liver fibrosis; FIB-4, fibrosis 4 index; LT, liver transplant; NCT, not-curative treatment (Sorafenib; TACE and best supportive care); not surv, patients opportunistically detected with HCC outside of surveillance program; OCT, other curative treatment for HCC (resection and ablation); surv, surveillance program of HCC in specialist care; VCTE, vibration controlled transient elastography.

Early detection of HCC and preventative management against DC were drivers of improved health outcomes with testing. Diagnostic algorithms with low specificity and high sensitivity were associated with more QALYs as a larger number of patients was monitored for HCC in specialist care, such as diagnostic algorithm 4 (FIB-4 only in primary care). Due to the higher number of patients in specialist care, this algorithm also yielded the highest cost.

Hence, with false positive patients in specialist care, resources will be allocated to patients with a low risk of developing HCC. Detailed results of disease progression and model results are available in Supplemental figures S1 to S3, http://links.lww.com/HC9/A332. Further details, including the decomposition of costs, can be found in Supplemental tables S12 to S14, http://links.lww.com/HC9/A332.

### Cost-effectiveness analysis

When evaluating all 13 patient management strategies, the no-testing strategy yielded the highest NHB at 12.438, indicating that no diagnostic algorithm had an incremental cost-effectiveness ratios below €50 000. The second highest NHB was observed with algorithm 5 (FIB-4 and ELF in primary care, VCTE in specialist care, and liver biopsy to patients with high or indeterminate value of VCTE), equivalent to an incremental cost-effectiveness ratios of ∼€181 000 compared with no testing. In terms of cost-effectiveness, all diagnostic algorithms but algorithms 5, 6, 8, and 4 were excluded by dominance or extended dominance (more expensive and with worse health outcomes) (Supplemental figure S4, http://links.lww.com/HC9/A332).

### Sensitivity analysis

Several one-way sensitivity analyses were conducted to understand how single model parameters in isolation impacted the base case results. The decrement in quality of life associated with the surveillance program in specialist care, the probability of an HCC diagnosis outside of specialist care, the interval of retesting, and the probability of DC patients outside of specialist care receiving liver transplants all had a minor impact on the results. Furthermore, a faster disease progression had a limited impact on the result and did not change the conclusions (Supplemental table S23, http://links.lww.com/HC9/A332). When a hypothetical treatment to slow down disease progression was applied to patients with known NAFLD, NASH, or fibrosis stage in primary care, several diagnostic algorithms yielded higher NHB than no-testing (Supplemental table S24–S26, http://links.lww.com/HC9/A332). Sensitivity analysis also showed that improving specificity at the cost of reduced sensitivity (higher cutoff) had a positive impact on NHB (though it did not change the conclusion of the study), Supplemental table S27, http://links.lww.com/HC9/A332. Details of the sensitivity analyses are available in Supplemental tables S15 to S27, http://links.lww.com/HC9/A332.

**FIGURE 3:**
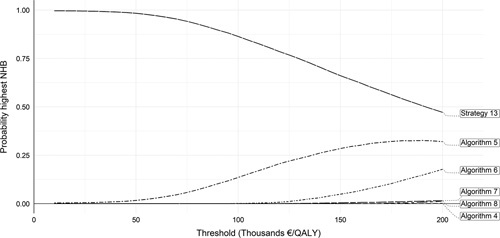
Cost-effectiveness acceptability curves.

The probabilistic sensitivity analysis indicated that at a threshold of €50 000, the no-testing strategy was associated with the highest NHB in 96% of the simulations; at a threshold of €100 000, this figure was ∼87.5%, see Figure 3. At a threshold of €100 000, algorithm 5 (FIB-4 and ELF in primary care, VCTE in specialist care with liver biopsy to patients with high or indeterminate values) yielded the highest NHB in 15% of the simulations.

## DISCUSSION

This study adds to previous literature by evaluating diagnostic algorithms with NITs for NAFLD in comprehensive health care management strategies spanning primary and secondary care. Two aspects of the result should be highlighted. First, our study questions the role of overall testing of the NAFLD population. Testing with NIT will improve health outcomes due to early detection and management of DC and HCC, but only at a cost per QALY that may be considered above conventional cost-effectiveness thresholds compared with a no-testing strategy in the base case results. Second, our study highlights the substantial uncertainties associated with evaluating comprehensive diagnostic management strategies, both in our application and in general. Depending on how these uncertainties resolve and if new treatments become available that may impact fibrosis progression, the cost-effectiveness of patient management strategies with comprehensive testing may improve.

Contrasting our findings with those of previous studies evaluating screening and diagnostic algorithms in NAFLD reveals both similarities and differences.^17–20^ One study found that a strategy with NAFLD fibrosis score in primary care followed by liver biopsy in indeterminate cases was cost-effective compared to liver biopsy for all patients,^20^ indicating that NITs have an important role in the management of patients with NAFLD. Our study adds to this knowledge and finds similar results when evaluating broader management strategies with NIT, as we find that a strategy of FIB-4 and ELF in primary care and VCTE and liver biopsy in specialist care performs well compared to other management strategies with active testing. An additional NIT test reduces the number of unnecessary liver biopsies and HCC screening cases by better-identifying patients with a risk of ESLD. The study by Tapper et al^20^ did not include a no-testing strategy and could, therefore, not evaluate the value of performing any testing compared with no testing.

Two previous studies found that screening high-risk patients for NAFLD was cost-effective,^17,18^ which somewhat contrasts our results. These studies modelled pharmacological treatments or weight loss interventions that, conditional on test findings, affected the fibrosis disease progression.^17–20^ However, the evidence of existing pharmacological treatments (which only indirectly target NAFLD) is ambiguous, and currently, there is no consensus in international guidelines.^13,46^ It has also been shown that long-term weight loss is challenging to maintain as patients only displayed a modest weight loss after 2 years,^47^ and its effect on the histological liver fibrosis stage is not clear.^48^ Hence, previous studies rely on uncertain treatment effects boosting the cost-effectiveness of the diagnostic tests, and our study indicates that if no such effect is applied, and the gain of testing lies in early detection and potential prevention of DC and early detection and better prognosis of HCC, the role of diagnostic testing is more uncertain as the benefits may not outweigh the additional costs.

The result from sensitivity analyses indicated that when a treatment to slow down disease progression was applied, our result was in line with previous studies and emphasized the importance of emerging treatments. However, to date, the evidence supporting such test-conditioned treatment effects is sparse. Our study therefore highlights a key question regarding diagnostic algorithm in the current management of NAFLD: it is crucial to provide evidence regarding the efficacy of treatments conditional on test results. If the primary gain is in the early detection of DC and HCC, in line with Swedish clinical practice and guidelines, many diagnostic algorithms cannot be considered cost-effective.

The emergence of pharmacological treatments for patients with NAFLD may change the playing field where future studies evaluating comprehensive patient management strategies incorporating both diagnostic algorithms and treatments are required. This study provides a framework for such evaluations. It should be emphasized that such studies must rely on treatment effects conditioned on test results; only then will improved treatment options have an impact on the effectiveness of testing. Additional tools to identify high-risk patients for HCC^49^ could also increase the effectiveness of identifying patients with NAFLD; perhaps this is a more promising avenue until further treatment options become available.

### Strengths and limitations

Some limitations of our model and results should be addressed. We evaluated VCTE in primary care, assuming no capacity constraints or learning curves. This may overestimate the value of VCTE if implemented in primary care before the method is used to full effect.

In addition, the transition probabilities used were derived from studies in tertiary care using primarily liver biopsy as a diagnostic tool, which could impose a bias and may not be generalizable for the whole NAFLD population. Indeed, the details of the natural course of NAFLD are not well established, and therefore modelling the disease progression is associated with large uncertainties. To analyze the impact of these uncertainties on the results, different scenarios were analyzed, and validation was carried out. Possible biases could lead to the model overestimating the value of identifying patients with NAFLD, but as our result support a no-testing strategy, it is likely that this aspect did not affect the implication of our result. It should be noted that even a modest treatment effect had a positive effect on the value of testing, which emphasizes the importance of developing treatments in the management of NAFLD. In addition, the lack of good data regarding several model inputs is prominent, and the emerging noninvasive diagnostics tools could, in the future, be used to monitor NAFLD patients to improve the understanding of disease progression. This may be an important aspect in the evaluation of emerging pharmacological treatments where the details of disease progression may be more important for the cost-effectiveness results.

A main strength of our study is that the comprehensive evaluation of several patient management strategies within a detailed disease progression model thoroughly illuminates the complexities associated with the decision problem at hand. The detailed presentation of the model anatomy with a breakdown of predicted clinical events, costs, and health outcomes should increase transparency and simplify validation, an aspect often lacking in previous studies.^26^ This should make comparisons with other model results easier as well as motivate further research regarding input parameters where data is still sparse. Also, the study highlights the importance of the general recommendation of including all relevant strategies in the economic evaluation,^23^ including, in this case, a no-testing strategy.

## CONCLUSION

If the benefits of noninvasive testing in patients with NAFLD are early detection and improved management of DC and HCC, this study questions the role of NIT. Testing will improve health outcomes due to early detection and management of DC and HCC, but at a cost per QALY above conventional cost-effectiveness thresholds compared with no testing. In the comparison between different diagnostic algorithms, a strategy with FIB-4 and ELF in primary care and VCTE and liver biopsy in specialist care is most promising in terms of cost-effectiveness. The study highlights the fact that the effectiveness and cost-effectiveness of diagnostic algorithms are highly dependent on conditional treatment effects, of which we have little evidence to date. If anything, our study calls to arms for further research regarding appropriate interventions conditional on fibrosis progression.

## Supplementary Material
