# Supplementary material for: Cost-effectiveness analysis of noninvasive tests to identify advanced fibrosis in non-alcoholic fatty liver disease
Source: Hepatol Commun. 2023 Jun 22;7(7):e00191. doi: 10.1097/HC9.0000000000000191 (PMC10289790; doi:10.1097/HC9.0000000000000191)
Supplement: Supplementary file 1 [file hc9-7-e00191-s001.pdf]

Cost-effectiveness analysis of non-invasive tests to identify advanced fibrosis in Non-Alcoholic Fatty Liver Disease.

Lina Gruneau[1*] M.Sc. (lina.gruneau@liu.se), Stergios Kechagias[2] MD, PhD (stergios.kechagias@liu.se), Per Sandström[3] MD, PhD (per.sandstrom@liu.se), Mattias Ekstedt[2,4] MD, PhD (mattias.ekstedt@liu.se), Martin Henriksson[1] PhD (martin.henriksson@liu.se)

[1]Center for Medical Technology Assessment, Department of Health, Medicine and Caring Sciences, Linköping University, Sweden

[2]Division of Diagnostics and Specialist Medicine, Department of Health, Medicine and Caring Sciences, Linköping University, Sweden

[3]Division of Surgery, Orthopedics and Oncology, Department of Biomedical and Clinical Sciences, Linköping University, Sweden

[4]Center for Medical Image Science and Visualization, Linköping University, Linköping, Sweden

*** Corresponding author:** Lina Gruneau, lina.gruneau@liu.se, +4613286856. Adress: Center for Medical Technology Assessment, Linköping University, SE-581 83 Linköping, 58183, Sweden. Fax: +4613288909

SUPPLEMENTARY MATERIAL

Table of contents

NAFLD disease progression model.......... 4
HCC treatments and prognosis.......... 6
Diagnostic algorithms.......... 8
Cost.......... 9
QALY-weights.......... 11
Validation.......... 12
Internal validation.......... 12
Cross validation.......... 12
Mortality.......... 12
DC and HCC.......... 13
External validation.......... 15
Mortality.......... 15
Results.......... 16
Predicted disease progression.......... 16
Mortality.......... 18
Health care costs.......... 19
Cost-effectiveness.......... 23
Sensitivity analysis.......... 24
QALY decrement.......... 24
Probability of HCC diagnosis outside the surveillance program.......... 24
Time interval of retesting.......... 24
Liver transplant.......... 24
Summary.......... 25
Progression.......... 25
Treatment response.......... 25
Diagnostic tests' characteristics.......... 26
References.......... 38

Supplementary figure 1. State occupancy in disease progression without any diagnostic algorithm applied ... 16
Supplementary figure 2. State occupancy regarding advanced disease for patient management strategies 4 and 5 and no testing ... 17
Supplementary figure 3. Number of patients in states representing transplantation, other curative and non-curative treatment, and liver transplant (both active treatment and post treatment) for patient management strategies 4 and 5 and no testing ... 17
Supplementary figure 4. Cost-effectiveness plane with frontier ... 23

Supplementary table 1. Transition probabilities and mortality hazard ratios for natural disease progression ... 4
Supplementary table 2. HCC parameters ... 6
Supplementary table 3. Costs ... 9
Supplementary table 4. Age-dependent QALY-weights general population ... 11
Supplementary table 5. Multiplier of decrement and QALY-weights ... 11
Supplementary table 6. Predicted 10-year survival based on starting stage ... 12
Supplementary table 7. Life expectancy based on starting stage ... 13
Supplementary table 8. 10-year cumulative incidence per 100 person years of DC based on starting stage ... 13
Supplementary table 9. 10-year cumulative incidence of HCC per 100 person years based on starting stage ... 14
Supplementary table 10. Predicted 10-year survival based on starting stage ... 15
Supplementary table 11. Predicted mortality and mean life expectancy ... 18
Supplementary table 12. Breakdown of undiscounted mean costs ... 20
Supplementary table 13. Breakdown of undiscounted mean costs in specialist care ... 21
Supplementary table 14. Undiscounted mean cost of diagnostic testing associated with the diagnostic algorithms ... 22
Supplementary table 15. Summary of NHB in selected one-way sensitivity analyses ... 25
Supplementary table 16. Diagnostic tests' characteristics ... 26
Supplementary table 17. One-way sensitivity analysis with a QALY decrement of 0.01 in specialist care ... 27
Supplementary table 18. One-way sensitivity analysis of no probability of diagnosis of HCC outside of surveillance program in BCLC stage 0/A-C ... 28
Supplementary table 19. One-way sensitivity analysis of no retesting ... 29
Supplementary table 20. One-way sensitivity analysis of retesting in 5-year intervals ... 30
Supplementary table 21. One-way sensitivity analysis of retesting in 10-year intervals ... 31
Supplementary table 22. One-way sensitivity analysis with a probability of liver transplant set to 0% for patients with DC not monitored in specialist care ... 32
Supplementary table 23. One-way sensitivity analysis of faster fibrosis progression reported as NHB ... 33
Supplementary table 24. One-way sensitivity analysis of treatment response in NAFLD-CC reported as NHB ... 34
Supplementary table 25. One-way sensitivity analysis of treatment response in NAFLD-F1 reported as NHB ... 35
Supplementary table 26. One-way sensitivity analysis of treatment response in F2-CC reported as NHB ... 36
Supplementary table 27. One-way sensitivity analysis of diagnostic testing reported as NHB ... 37

NAFLD disease progression model

The details of the disease progression model depicting the natural course of NAFLD are described below. Note that all transitions described below refer to annual transitions.

NAFLD: NAFLD patients could progress to NASH, NASH F1, NASH F2, NASH F3, HCC. Mortality rate was conditional on age.

NASH: NASH patients could regress to NAFLD, and progress to NASH F1, NASH F2, NASH F3, CC, HCC. Mortality rate was conditional on age.

NASH F1: NASH F1 patients could regress to NAFLD, NASH, and progress to NASH F2, NASH F3, CC, HCC. Mortality was conditional on age.

NASH F2: NASH F2 patients could regress to NAFLD, NASH, NASH F1, and progress to NASH F3, CC, HCC. Mortality rate was conditional on age.

NASH F3: NASH F3 patients could regress to NASH F1, NASH F2, and progress to CC, HCC. Mortality rate was conditional on age.

CC: CC patients could regress to NASH F1, NASH F2, NASH F3 and progress to DC, HCC. Mortality rate was conditional on age.

DC: DC patients could transition to HCC. DC patients could be treated with liver transplant. Mortality rate was conditional on state occupancy.

HCC patients: Patients surviving a cycle (year) in any of the BCLC stages transitioned to the next stage at the end of the cycle. Hence, a patient only resided in each stage for one cycle until reaching the BCLC stage D where they stayed as long as they survived in the model. Patient could receive liver transplant, other curative treatment or non-curative treatment. Details of HCC treatments are described in a separate section below. Mortality was conditional on BCLC stage.

Detailed transition probabilities for progression and regression between health states can be found in Supplementary table 1. Note that NASH F1, NASH F2, NASH F3 will be called F1, F2, and F3 henceforth.

Supplementary table 1. Transition probabilities and mortality hazard ratios for natural disease progression

Parameter	Value	Distribution	Alpha	Beta	Source
**Mortality**					
HR NAFLD (against general population)	1.340	Lognormal	0.148		[1]
HR NASH (against general population)	1.340	Lognormal	0.148		[1]
HR F1 (against NAFLD/NASH)	1.580	Lognormal	0.146		[2]
Hr F2 (against F1)	1.590	Lognormal	0.214		[2]
HR F3 (against F2)	1.380	Lognormal	0.229		[2]
HR CC (against F3)	1.840	Lognormal	0.281		[2]
**From NAFLD**					
NAFLD to NASH	0.047	Beta	8.002	161.033	[3,4]
NAFLD to F1	0.004	Beta	8.414	2233.794	[3,4]
NAFLD to F2	0.008	Beta	8.373	1027.590	[3,4]
NAFLD to HCC	0.0003	Beta	11.960	39853.278	[5]
**From NASH**					

NASH to NAFLD	0.050	Beta	4.108	78.505	[3,4]
NASH to F1	0.050	Beta	34.146	644.076	[3,4]
NASH to F2	0.003	Beta	35.883	10490.512	[3,4]
NASH to F3	0.007	Beta	35.737	4831.691	[3,4]
NASH to CC	0.002	Beta	35.941	19544.800	[3,4]
NASH to HCC	0.0003	Beta	11.960	39853.278	[5]
**From F1**					
F1 to NASH	0.020	Beta	9.501	471.515	[3,4]
F1 to NAFLD	0.005	Beta	9.660	1946.542	[3,4]
F1 to F2	0.053	Beta	25.602	454.395	[3,4]
F1 to F3	0.012	Beta	26.771	2248.509	[3,4]
F1 to CC	0.002	Beta	27.057	17220.201	[3,4]
F1 to HCC	0.004	Beta	99.596	24799.404	[6]
**From F2**					
F2 to NASH	0.007	Beta	5.890	873.845	[3,4]
F2 to NAFLD	0.003	Beta	5.916	2056.045	[3,4]
F2 to F1	0.038	Beta	5.671	142.561	[3,4]
F2 to F3	0.045	Beta	71.942	1523.932	[3,4]
F2 to CC	0.011	Beta	74.525	6538.127	[3,4]
F2 to HCC	0.004	Beta	99.596	24788.404	[6]
**From F3**					
F3 to F1	0.015	Beta	12.393	793.551	[3,4]
F3 to F2	0.062	Beta	11.765	179.519	[3,4]
F3 to CC	0.044	Beta	140.365	3049.561	[3,4]
F3 to HCC	0.0053	Beta	24.862	4666.119	[6]
**From CC**					
CC to F3	0.046	Beta	4.890	101.614	[3,4]
CC to HCC	0.014	Beta	98.637	7207.771	[6]
CC to DC (undetected)	0.060	Beta	90.218	1413.409	[17*]
CC to DC (detected)	0.042	Log normal (HR 0.7)	0.150		Assumption[7,17*]
**From DC**					
DC to HCC	0.026	Beta	11.373	426.062	[6]
DC to liver transplant	0.008	Beta	65.487	8606.579	[8]
DC to death	0.202	Beta	152.137	601.016	[9]
**From HCC (not in SC)**					
HCC 0/A to death	0.280	Beta	3199.720	8227.851	[6]
HCC B to death	0.504	Beta	9.967	9.809	[10]
HCC C to death	0.750	Beta	31.216	10.405	[10]
HCC D to death	0.890	Beta	43.846	5.419	[10]
**From HCC with CC (not in SC)**					
CCHCC 0/A to death	0.280	Beta	3199.720	8227.851	[6]
CCHCC B to death	0.504	Beta	9.967	9.809	[10]
CCHCC C to death	0.750	Beta	31.216	10.405	[10]
CCHCC D to death	0.890	Beta	43.846	5.419	[10]
**From HCC with DC**					
DCHCC to death	0.280	Beta	3199.720	8227.851	[6]

All values except hazard ratios are annual transition probabilities. HR= Hazard Ratio. F= Fibrosis. CC= Compensated Cirrhosis. DC=Decompensated Cirrhosis. SC=specialist care.

HCC treatments and prognosis

HCC treatments included in the model were categorized as liver transplant, other curative (estimated from patients receiving ablation and resection), and non-curative (estimated from patients receiving TACE, sorafenib or palliative care). Treatment affected mortality, quality of life and costs. Liver transplant and other curative treatment were defined as a one-time event during the cycle they occurred. Patients were assumed to receive post operative care in proceeding years with costs, survival and quality of life reflecting the post treatment prognosis. Post non-curative treatment was defined as palliative care and was modelled with annual costs, survival and quality of life reflecting the annual prognosis of these patients.

In the surveillance program in specialist care, the test for identifying HCC was assumed to be perfect. Hence, in the same cycle that patients developed HCC they were detected by the surveillance program and received treatment. Therefore, patients detected with the surveillance program never progressed beyond BCLC 0/A and thus received treatment conditional on this state. Outside of the surveillance program the probability of being diagnosed with HCC was conditional on BCLC stage. The probability of identification was based on assumptions reflecting both chance and symptoms. Furthermore, the proportions treated with transplantation, other curative and non-curative HCC treatment were conditional on BCLC stage where the probability of other curative treatment (resection and ablation) decreased with more severe BCLC stage.

Mortality rates were conditional on treatment and were estimated using registry data from a retrospective study on 3308 patients.[28*] Mortality rates for the initial treatments as well as long-term post treatment prognosis were not differentiated based on BCLC stage. The estimated probabilities of being diagnosed with HCC at different stages of the disease as well as the estimated proportions of patients receiving transplant, other curative treatment and non-curative treatment by HCC-stage can be found in Supplementary table 2.

Supplementary table 2. HCC parameters

	Value	Distribution	Alpha	Beta	Source
**Annual probability of being diagnosed outside of surveillance program**					
HCC 0/A	0.100	Beta	22	202	Assumption
HCC B	0.150	Beta	21	120	Assumption
HCC C	0.250	Beta	19	56	Assumption
HCC D	1				Assumption
**Treatment according to HCC-stage (proportion of patients in each treatment category)**					
Without cirrhosis					
HCC 0/A					
Liver transplant	0.120	Dirichlet	42		[28*]
Other curative treatment	0.700	Dirichlet	238		[28*]
Non-curative treatment	0.180	Dirichlet	62		[28*]
HCC B					
Liver transplant	0.060	Dirichlet	3		[28*]
Other curative treatment	0.280	Dirichlet	13		[28*]
Non-curative treatment	0.680	Dirichlet	31		[28*]
HCC C					
Liver transplant	0.040	Dirichlet	47		[28*]
Other curative treatment	0.170	Dirichlet	195		[28*]

Non-curative treatment	0.780	Dirichlet	876		[28*]
HCC D					
Liver transplant	0.060	Dirichlet	28		[28*]
Other curative treatment	0.020	Dirichlet	7		[28*]
Non-curative treatment	0.920	Dirichlet	399		[28*]
With cirrhosis					
CCHCC 0/A					
Liver transplant	0.060	Dirichlet	3		[28*]
Other curative treatment	0.280	Dirichlet	13		[28*]
Non-curative treatment	0.680	Dirichlet	31		[28*]
HCC B					
Liver transplant	0.040	Dirichlet	47		[28*]
Other curative treatment	0.170	Dirichlet	195		[28*]
Non-curative treatment	0.780	Dirichlet	876		[28*]
HCC C					
Liver transplant	0.060	Dirichlet	28		Assumption [28*]
Other curative treatment	0.020	Dirichlet	7		Assumption [28*]
Non-curative treatment	0.920	Dirichlet	399		Assumption [28*]
HCC D					
Liver transplant	0.060	Dirichlet	28		Assumption [28*]
Other curative treatment	0.020	Dirichlet	7		Assumption [28*]
Non-curative treatment	0.920	Dirichlet	399		Assumption [28*]
HCC with DC					
Liver transplant	0.100	Dirichlet	10		Assumption
Other curative treatment	0				Assumption
Non-curative treatment	0.900	Dirichlet	90		Assumption
**Annual mortality probability conditional on treatment**					
Liver transplant	0.059	Beta	37.560	594.903	[28*]
Other curative treatment	0.170	Beta	208.985	1015.158	[28*]
Non-curative treatment	0.480	Beta	506.590	542.638	[28*]

CC= Compensated Cirrhosis. DC=Decompensated Cirrhosis. HCC=Hepatocellular Carcinoma.

Diagnostic algorithms

Details of how the diagnostic algorithms impact the patient management trajectory is described per health state below.

NAFLD, NASH, F1, F2: True negative patients (i.e., correctly assessed not to have F3 or CC) were retained in primary care and re-tested every 3 years in the base case analysis. Patients with a false positive diagnosis of advanced disease (F3 or CC) were retained in specialist care where they were screened for HCC. These patients could not be re-categorized as true negatives in the model as HCC-screening would not detect the incorrectly diagnosed advanced disease. Disease progression was not affected by being in primary or specialist care.

F3: Patients with F3 could be correctly diagnosed with advanced disease and be retained in specialist care where they were screened for HCC. F3 differed from previous health states as non-invasive test threshold values diagnosed F3 patients as true positive. However, diagnosing with liver biopsy is focused on ruling in cirrhosis. Therefore, in diagnostic algorithms only containing non-invasive tests F3 was considered true positive and correctly retained in specialist care but in algorithms combining NIT with liver biopsy patients with F3 were ruled out. False negative patients (i.e., incorrectly assessed to not to have advanced disease) were retained in primary care and re-tested every 3 years in the base case analysis. Disease progression was not affected by being in primary or specialist care.

CC: Patients with CC could be correctly diagnosed with advanced disease and be retained in specialist care where they were screened for HCC. Patients with CC in specialist care had a lower probability of developing DC due to preventative intervention. Therefore, patients with CC outside of specialist care had a higher probability of developing DC. False negative patients (i.e., incorrectly assessed to not to have advanced disease) were retained in primary care and re-tested every 3 years in the base case analysis. These patients were not differentiated from patients progressing in their disease. Hence, a patient with CC in primary care with an initial false negative assessment was not differentiated from a patient in primary care that had subsequently progressed into CC.

Cost

All applied cost can be found in Supplementary table 3 and table 2 in the main manuscript. In the no-testing strategy patients were assumed to be in primary care without any scheduled testing and did therefore not incur any diagnostic costs. The cost of primary care was based on assumed number of physician visits. The number of visits was based on information from a study on outpatient care visits [42*] and clinical experts as data was lacking.

The cost in specialist care for false positive patients was based upon one physician appointment, one nurse appointment, two computed tomography scans per year in the surveillance program for HCC as well as one VCTE for monitoring of DC per year, in accordance with Swedish guidelines.[11] Patients with detected CC received above-described surveillance and in addition gastroscopy every third year. If patients transitioned to DC, they received treatment. The probability of different events for decomposing.[12] (e.g., ascites or varices etc) was calculated and the cost for their treatment was based on local data.

The cost of HCC was based on the different treatments received. Patient in the post liver transplant state received two physician appointments, four nurse appointments and immunosuppressive treatment each year.[13] For patients treated with other curative treatment, post treatment surveillance was assumed to be one doctor's appointment, one nurse appointment and one computed tomography scan per year. Post non-curative treatment cost was assumed to be palliative care.

Patients developing DC outside of specialist were assumed to incurring a one-time cost of diagnostic work up consisting of two physician visits in specialist care as well as VCTE and computed tomography scan. The same cost applied to opportunistically detected HCC cases outside of surveillance (see section HCC treatment and prognosis).

Supplementary table 3. Costs

Parameter	Value (€)	Distribution	Scale	Shape	Source
**Primary care**					
NAFLD	269	Gamma	7.539	35.630	Assumption [37*,42*]
NASH	269	Gamma	7.539	35.630	Assumption [37*,42*]
F1	288	Gamma	26.852	10.724	Assumption [37*,42*]
F2	288	Gamma	20.264	14.211	Assumption [37*,42*]
F3	398	Gamma	26.656	14.915	Assumption [37*,42*]
CC	666	Gamma	14.221	46.843	Assumption [37*,42*]
CCHCC 0/A	666	Gamma	14.221	46.843	Assumption [37*,42*]
CCHCC B	666	Gamma	14.221	46.843	Assumption [37*,42*]
CCHCC C	666	Gamma	14.221	46.843	Assumption [37*,42*]
CCHCC D	666	Gamma	14.221	46.843	Assumption [37*,42*]
HCC 0/A	398	Gamma	26.656	14.915	Assumption [37*,42*]
HCC B	398	Gamma	26.656	14.915	Assumption [37*,42*]
HCC C	398	Gamma	26.656	14.915	Assumption [37*,42*]
HCC D	398	Gamma	26.656	14.915	Assumption [37*,42*]
**Specialist care**					
NAFLD	2120	Gamma	170.834	12.409	Local data [37*]
NASH	2120	Gamma	170.834	12.409	Local data [37*]
F1	2120	Gamma	170.834	12.409	Local data [37*]
F2	2120	Gamma	170.834	12.409	Local data [37*]
F3	2120	Gamma	170.834	12.409	Local data [37*]
CC	3137	Gamma	139.112	22.547	Local data [37*]

DC (1st year)	14744	Gamma	96.040	153.515	Local data [37*]
DC (2nd year and onwards)	3551	Gamma	93.655	37.921	Local data [37*]
Liver transplant (1st year)	76374	Gamma	96.040	795.277	Local data [37*]
Other curative treatment (1st year)	10456	Gamma	96.040	108.875	Local data[11,14]
Non-curative treatment (1st year)	8417	Gamma	96.040	87.638	Local data[15–17]
Post liver transplant	4355	Gamma	42.904	101.512	Local data [18,19, 37*]
Post other curative treatment	1717	Gamma	112.086	15.320	Assumption, local data [37*]
Post non-curative treatment	8352	Gamma	96.040	86.961	[15]
Diagnosing of HCC (not in surveillance program)	1806	Gamma	79.861	22.614	Assumption, local data [37*]
Diagnosing of DC (not previously known CC)	1806	Gamma	79.861	22.614	Assumption, local data [37*]

All cost except diagnosing of HCC and DC are expressed as annual costs. F= Fibrosis. CC= Compensated Cirrhosis. DC=Decompensated Cirrhosis. Other curative treatment=resection or ablation. Non-curative treatment= TACE, sorafenib or best supportive care.

QALY-weights

Age-related QALY-weights for all states in the model were derived from secondary sources. Younossi et al.[6] who relied on the quality-of-life assessment from Sayiner et al.[20] was used for disease-specific weights. The reported QALY-weights were transformed to decrements with associated uncertainty estimates to facilitate the probabilistic analysis. Age-adjusted QALY-weights for the general population were retrieved from a Swedish study by Teni et al.[21] see Supplementary table 4. Patients with NAFLD or NASH were assumed to have the same QALY-weight as the general population and hence the multiplier for these two groups from the age-adjusted general population values was set to 1. The QALY-weights for curative treatment were based on assumptions as data was lacking in the literature. QALY-weights for HCC in BCLC stages were not available and they were assumed to be the same as those for patients with chronic hepatis-B related HCC.[22] Distributions representing the uncertainty around the multipliers of decrement were defined as beta distributions based on available data, Supplementary table 5. Note that the QALY-weights reported in Supplementary table 5 are for 55-year-old patients.

Supplementary table 4. Age-dependent QALY-weights general population

Age	QALY-weight	Source
50-54	0.903	21
55-59	0.901	21
60-64	0.904	21
65-69	0.912	21
70-74	0.904	21
75-79	0.887	21
80-84	0.858	21
85-89	0.824	21
90-94	0.783	21
95-104	0.718	21

Supplementary table 5. Multiplier of decrement and QALY-weights

	QALY-weights*	Multiplier	QALY-weight multiplier is applied to	Alpha	Beta	Source
NAFLD	0.901	1	General population			6
NASH	0.901	1	General population			6
F1	0.865	0.961	NASH	13.041	0.536	6
F2	0.794	0.918	F2	30.425	2.725	6
F3	0.759	0.955	F2	19.221	0.901	6
CC	0.630	0.830	F3	84.809	17.409	6
DC	0.561	0.891	CC	78.696	9.650	6
DCHCC	0.533	0.968	HCC 0/A	102.322	3.384	Assumption[6]
HCC 0/A	0.551	0.875	CC	322.923	46.273	22
HCC B	0.533	0.968	HCC 0/A	102.322	3.384	22
HCC C	0.517	0.969	HCC B	96.403	3.135	22
HCC D	0.517	0.969	Same as HCC B	96.403	3.135	22
Liver transplant (1st year)	0.572	0.908	CC	142.608	14.467	23
Other curative treatment (1st year)	0.504	0.800	CC	141.422	35.356	Assumption
Non-curative treatment (1st year)	0.315	0.500	CC	49.500	49.500	24
Post liver transplant (2nd year onwards)	0.605	0.961	CC	55.489	2.280	23
Post- other curative treatment	0.535	0.850	CC	34.976	6.172	Assumption
Post- non-curative treatment	0.315	0.500	CC	49.500	49.500	24

*QALY-weights are expressed for 55-year-old patients. Alpha and Beta are distributional parameters of the beta distribution. F= Fibrosis. CC= Compensated Cirrhosis. DC=Decompensated Cirrhosis.

Validation

Internal validation

We checked for internal validity by assessing that the model and mathematical calculations were consistent and free from computational errors.[25,26] Such tests ranged from summing all patients in one cycle to check that the cohort was intact throughout the entire analysis time to isolating particular features of the model to validate that the diagnostic algorithms did not change anything else than moving patients from primary care to specialist care without changing their disease status. Sensitivity analyses applying extreme values were conducted to detect logical flaws, e.g., checking if 0 sensitivity and specificity of tests provided the same number of detected individuals as in the no-testing strategy.

Cross validation

Mortality

As part of validation, predicted 10-year survival and life expectancy for 49-year-old patients were compared with against previously conducted Markov-models of disease progression for patients with NAFLD, see Supplementary table 6. Compared to Chhatwal et al.[27] our results are similar. However, for patients with CC at baseline our model predicts higher overall survival than Chhatwal et al.[27] and Younossi et al.[6] but our model differs from the previous models in that we modelled advanced liver disease differently. This could explain the difference in the results. Overall, our predicted survival is in line with previous effort modelling disease progression.

Supplementary table 6. Predicted 10-year survival based on starting stage

			NAFLD	F0	F1	F2	F3	CC
Previous studies								
Chhatwal et al.[27]			0.941 (92.7–94.6)	0.929 (89.8–94.1)	0.922 (88.4–93.9)	0.887 (82.3–91.9)	0.814 (72.9–86.9)	0.513 (44.2–59.4)
Younossi et al.[6]				-	-	0.859	0.736	0.538
Our model								
Range			0.964	0.955-0.956	0.922-0.919	0.889-0.893	0.844-0.854	0.613-0.648
Strategy number	Primary care	Specialist care						
1	FIB-4	VCTE. Liver biopsy	0.964	0.955	0.919	0.890	0.845	0.646
2	FIB-4	Liver biopsy	0.964	0.955	0.919	0.890	0.846	0.648
3	FIB-4	VCTE. Liver biopsy*	0.964	0.955	0.920	0.891	0.851	0.646
4	FIB-4		0.964	0.956	0.922	0.893	0.854	0.648
5	FIB-4. ELF	VCTE. Liver biopsy	0.964	0.955	0.919	0.890	0.845	0.643
6	FIB-4. ELF	VCTE. Liver biopsy*	0.964	0.955	0.919	0.890	0.850	0.643
7	FIB-4. ELF	Liver biopsy	0.964	0.955	0.919	0.890	0.845	0.645
8	FIB-4. ELF		0.964	0.955	0.920	0.891	0.852	0.645
9	FIB-4. VCTE	Liver biopsy	0.964	0.955	0.919	0.890	0.845	0.646
10	FIB-4. VCTE	Liver biopsy*	0.964	0.955	0.920	0.891	0.851	0.646
11	VCTE	Liver biopsy	0.964	0.955	0.919	0.890	0.846	0.650
12	VCTE	Liver biopsy*	0.964	0.956	0.921	0.892	0.854	0.650
13	No testing	No testing	0.964	0.955	0.919	0.889	0.844	0.613

F= Fibrosis. CC= Compensated Cirrhosis.

Chhatwal et al.[27] also presented life expectancy for 49-year-olds. Our results are compared with this study in Supplementary table 7. Our model seems to predict higher life expectancy for patients with CC

at baseline compared to Chhatwal et al.[27] however such differences can be due to difference in modelling of advanced liver disease. As previously mentioned, we modelled HCC in accordance with BCLC stages which is different from the comparator study.

Supplementary table 7. Life expectancy based on starting stage

			F0	F1	F2	F3	CC
Previous studies							
Chhatwal et al.[27]			25.3 (20.1-29.8)	25.1 (20.1-29.4)	23.6 (18.3-28.2)	21.1 (15.6-26.3)	13.8 (10.3-17.6)
Our model							
Range			28.539-28.669	25.622-25.835	23.642-23.898	21.317-21.732	14.529-15.423
Strategy number	Primary care	Specialist care					
1	FIB-4	VCTE. Liver biopsy	28.555	25.649	23.703	21.445	15.371
2	FIB-4	Liver biopsy	28.556	25.651	23.707	21.452	15.417
3	FIB-4	VCTE. Liver biopsy*	28.600	25.719	23.785	21.658	15.377
4	FIB-4		28.669	25.835	23.898	21.732	15.423
5	FIB-4. ELF	VCTE. Liver biopsy	28.554	25.647	23.699	21.437	15.316
6	FIB-4. ELF	VCTE. Liver biopsy*	28.573	25.678	23.743	21.607	15.323
7	FIB-4. ELF	Liver biopsy	28.555	25.649	23.703	21.444	15.362
8	FIB-4. ELF		28.603	25.724	23.791	21.674	15.368
9	FIB-4. VCTE	Liver biopsy	28.555	25.649	23.703	21.445	15.371
10	FIB-4. VCTE	Liver biopsy*	28.600	25.719	23.785	21.658	15.377
11	VCTE	Liver biopsy	28.555	25.650	23.708	21.455	15.445
12	VCTE	Liver biopsy*	28.647	25.795	23.860	21.721	15.449
13	No testing	No testing	28.539	25.622	23.642	21.317	14.529

F= Fibrosis. CC= Compensated Cirrhosis.

DC and HCC

An overall lack of reported clinical outcomes in modelling studies precludes comparison of expected DC and HCC incidence with many studies. Chhatwal et al.[27] reported 10-year cumulative incidence per 100-person years for developing DC and HCC by fibrosis stage (see Supplementary table 8). Applied transition probabilities determine the cumulative incidence. Of note, the probability of regression for patients with CC in our model was 0.046 compared with 0.086 in Chhatwal et al.[27] This likely explains the difference in DC incidence between the studies.

Supplementary table 8. 10-year cumulative incidence per 100 person years of DC based on starting stage

			NAFLD	F0	F1	F2	F3	CC
Previous studies								
Chhatwal et al.[27]			0.007 (0.003-0.01)	0.08 (0.05-0.1)	0.15 (0.10-0.25)	0.7 (0.51-1.0)	2.7 (2.1-3.4)	17.2 (15.9-18.6)
Our model								
Range			0.066-0.080	0.320-0.384	0.489-0.589	1.633-1.959	4.118-5.287	26.557-32.749
Strategy number	Primary care	Specialist care						
1	FIB-4	VCTE. Liver biopsy	0.073	0.347	0.533	1.768	4.764	26.916
2	FIB-4	Liver biopsy	0.072	0.344	0.529	1.753	4.723	26.558

3	FIB-4	VCTE. Liver biopsy*	0.071	0.340	0.522	1.734	4.338	26.915
4	FIB-4		0.066	0.320	0.489	1.633	4.118	26.557
5	FIB-4. ELF	VCTE. Liver biopsy	0.073	0.350	0.538	1.785	4.810	27.335
6	FIB-4. ELF	VCTE. Liver biopsy*	0.073	0.347	0.533	1.771	4.478	27.334
7	FIB-4. ELF	Liver biopsy	0.073	0.348	0.534	1.771	4.772	26.986
8	FIB-4. ELF		0.071	0.340	0.521	1.734	4.292	26.985
9	FIB-4. VCTE	Liver biopsy	0.073	0.347	0.533	1.768	4.764	26.916
10	FIB-4. VCTE	Liver biopsy*	0.071	0.340	0.522	1.734	4.338	26.915
11	VCTE	Liver biopsy	0.072	0.342	0.526	1.744	4.698	26.346
12	VCTE	Liver biopsy*	0.068	0.326	0.499	1.664	4.143	26.347
13	No testing	No testing	0.080	0.384	0.589	1.959	5.287	32.749

. F= Fibrosis. CC= Compensated Cirrhosis. DC=Decompensated Cirrhosis.

10-year cumulative incidence of HCC is higher for all fibrosis stages in our model compared to Chhatwal et al.[27] see Supplementary table 9. This is explained by the fact that we have allowed patients in the early stages of fibrosis to develop HCC.

Supplementary table 9. 10-year cumulative incidence of HCC per 100 person years based on starting stage

			NAFLD	F0	F1	F2	F3	CC
Previous studies								
Chhatwal et al.[27]			0.002 (0.0003-0.005)	0.03 (0.02-0.05)	0.06 (0.03-0.09)	0.3 (0.2-0.4)	1.1 (0.9-1.4)	7.9 (7.4-8.7)
Our model								
Range			0.454-0.485	0.956-0.986	2.992-3.393	3.433-3.838	4.595-5.143	9.019-10.304
Strategy number	Primary care	Specialist care						
1	FIB-4	VCTE. Liver biopsy	0.455	0.956	2.993	3.435	4.606	9.124
2	FIB-4	Liver biopsy	0.455	0.956	2.993	3.435	4.605	9.018
3	FIB-4	VCTE. Liver biopsy*	0.455	0.956	2.993	3.435	4.599	9.125
4	FIB-4		0.454	0.956	2.992	3.433	4.595	9.019
5	FIB-4. ELF	VCTE. Liver biopsy	0.455	0.956	2.993	3.436	4.607	9.240
6	FIB-4. ELF	VCTE. Liver biopsy*	0.455	0.956	2.993	3.436	4.601	9.240
7	FIB-4. ELF	Liver biopsy	0.455	0.956	2.993	3.435	4.606	9.144
8	FIB-4. ELF		0.455	0.956	2.993	3.435	4.598	9.145
9	FIB-4. VCTE	Liver biopsy	0.455	0.956	2.993	3.435	4.606	9.124
10	FIB-4. VCTE	Liver biopsy*	0.455	0.956	2.993	3.435	4.599	9.125
11	VCTE	Liver biopsy	0.455	0.956	2.992	3.435	4.605	8.952
12	VCTE	Liver biopsy*	0.455	0.956	2.992	3.434	4.595	8.953
13	No testing	No testing	0.485	0.986	3.393	3.838	5.143	10.304

F= Fibrosis. CC= Compensated Cirrhosis.

External validation

Mortality

In a meta-analysis of biopsy-proven NAFLD cohort studies 10-year survival was reported.[28] These results were compared with the predicted mortality from our model for patients aged 51, see Supplementary table 10. The meta-analysis reported result for categories of fibrosis stage, and an assumption regarding the distribution among fibrosis stages had to be made to enable a comparison. Overall, our results are in line with Ng et al.[28] but mortality differs between the strategies. For example, our model predicted a 10-year survival for CC patients at 59.1-62.5% while Ng et al.[28] presented a survival of 58.5%. Comparison in earlier fibrosis stages should be made with caution due to assumptions but seems to be in line with our result.

Supplementary table 10. Predicted 10-year survival based on starting stage

			F0-F2*	F2-F4*	F3-CC*	CC
Previous studies						
Ng et al. [28]			0.923	0.707	0.678	0.585
Our model						
Range			0.924-0.926	0.707-0.729	0.685-0.709	0.591-0.625
Strategy	Primary care	Specialist care				
1	FIB-4	VCTE. Liver biopsy	0.924	0.724	0.704	0.623
2	FIB-4	Liver biopsy	0.924	0.725	0.705	0.625
3	FIB-4	VCTE. Liver biopsy*	0.924	0.726	0.706	0.623
4	FIB-4		0.926	0.729	0.709	0.625
5	FIB-4. ELF	VCTE. Liver biopsy	0.924	0.723	0.702	0.620
6	FIB-4. ELF	VCTE. Liver biopsy*	0.924	0.724	0.704	0.620
7	FIB-4. ELF	Liver biopsy	0.924	0.724	0.704	0.622
8	FIB-4. ELF		0.924	0.726	0.706	0.622
9	FIB-4. VCTE	Liver biopsy	0.924	0.724	0.704	0.623
10	FIB-4. VCTE	Liver biopsy*	0.924	0.726	0.706	0.623
11	VCTE	Liver biopsy	0.924	0.726	0.706	0.627
12	VCTE	Liver biopsy*	0.925	0.729	0.710	0.627
13	No testing	No testing	0.924	0.707	0.685	0.591

*Proportion of fibrosis stage is based on assumption from the cohort in Ng et al.[28]: *F0-F2:* F0: 54.5%, F1: 29.5%, F2: 16%. *F2-CC:* F2: 12%, F3: 35%, CC: 53%. *F3-CC:* F3: 40%, CC: 60%. F= Fibrosis. CC= Compensated Cirrhosis.

Results

Predicted disease progression

The Markov model trace in Supplementary figure 1 shows the proportion of patients of the cohort in each state of the model for every year of the analysis when no diagnostic algorithm is applied. At year zero the distribution of the cohort across states (prevalence) can be seen, and the disease progression over time thus represents our best estimate of the natural course of the disease. A further breakdown of predicted advanced liver disease and treatment (liver transplant, other curative treatment, and non-curative treatment) is illustrated in Supplementary figure 2 and 3. Note that liver transplant given to patients with DC is included in this graph. Also note that figures represent the number of patients from a cohort of 10 000 individuals.

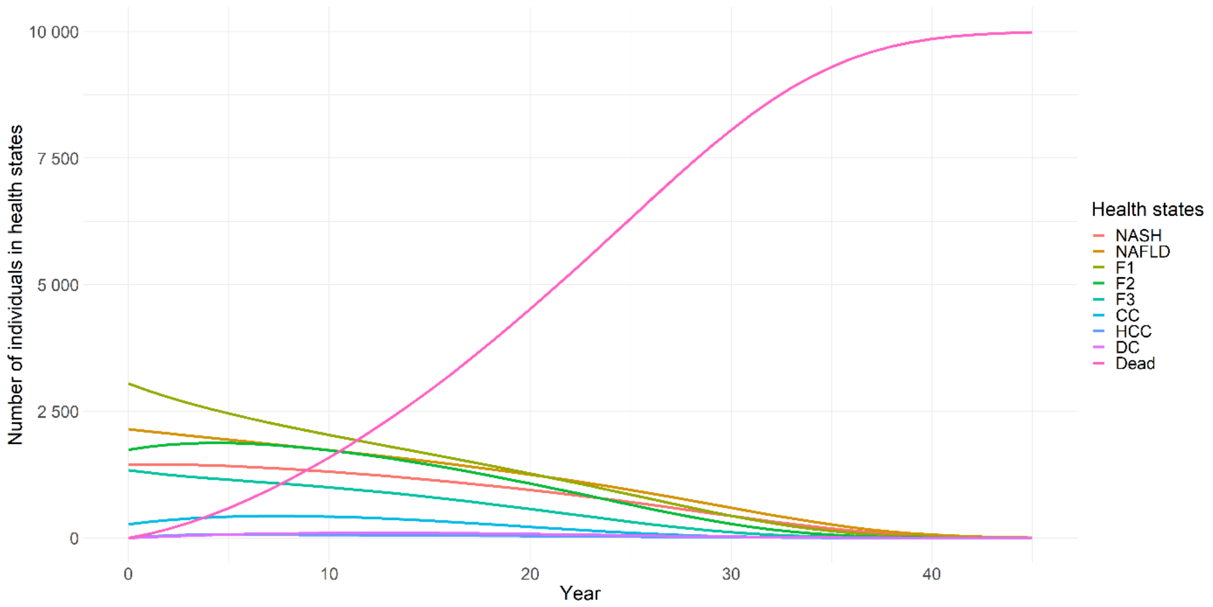

Supplementary figure 1. State occupancy in disease progression without any diagnostic algorithm applied

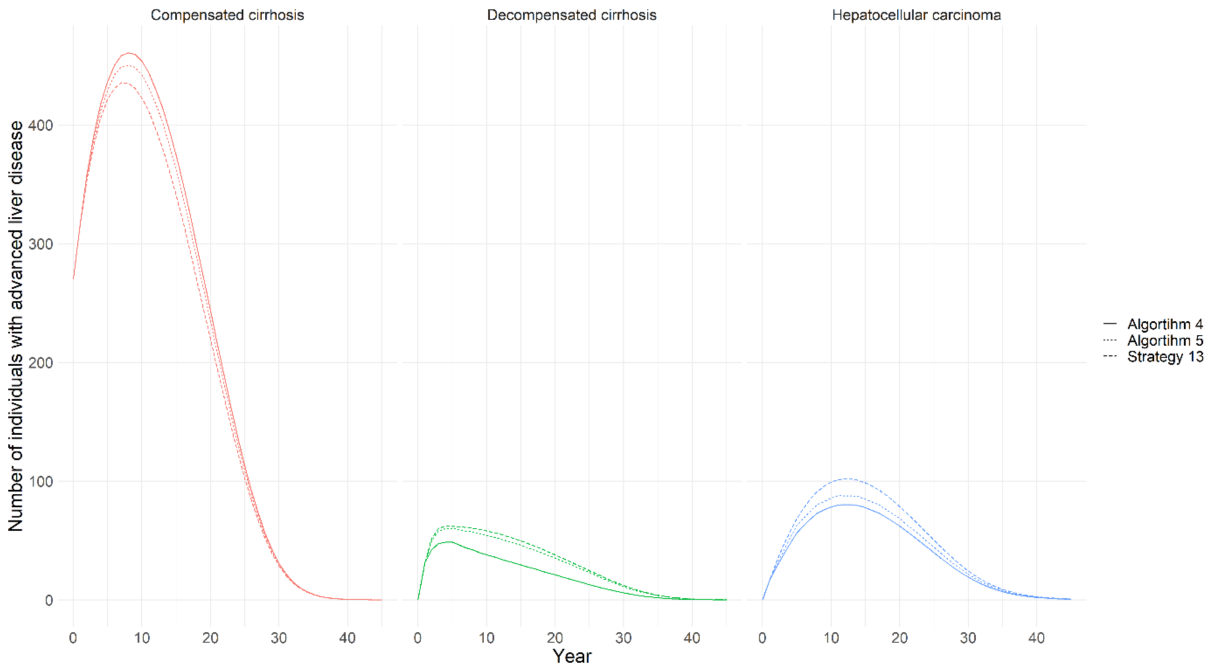

Supplementary figure 2. State occupancy regarding advanced disease for patient management strategies 4 and 5 and no testing

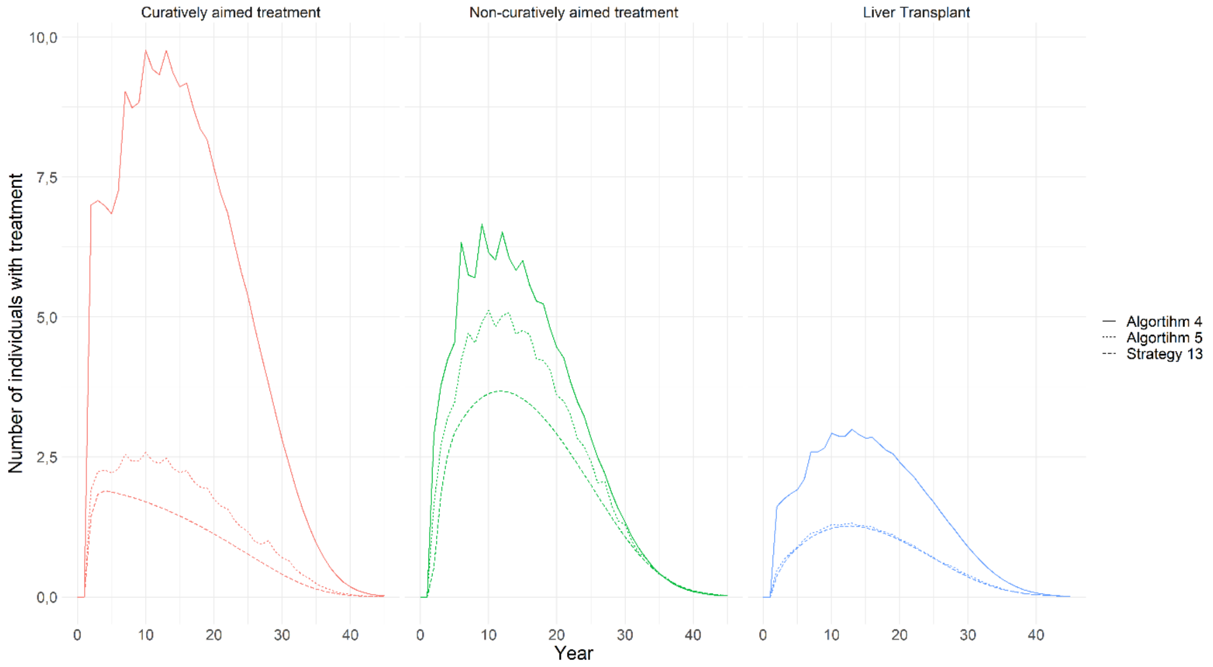

Supplementary figure 3. Number of patients in states representing transplantation, other curative and non-curative treatment, and liver transplant (both active treatment and post treatment) for patient management strategies 4 and 5 and no testing

Mortality

Predicted mortality at different follow-up times in the model, as well as mean survival are presented in Supplementary table 11. To facilitate comparison with registry data and clinical experience results were left undiscounted. The model predicted that mean survival (life-expectancy) for a 55-year-old cohort was around 21 years. Ater 5, 15, and 20 years approximately 431, 2598, and 4153 patients, respectively, had died in the model. Note that exact figures depend on the evaluated strategy.

Supplementary table 11. Predicted mortality and mean life expectancy

**Patient management strategies**					**Cases per 10 000**		**Per patient mean**
Strategy	Primary care	Specialist care	1-year mortality	5-year mortality	15-year mortality	20-year mortality	Life years
1	FIB-4	VCTE. Liver biopsy	77.553	433.491	2608.598	4165.385	21.455
2	FIB-4	Liver biopsy	77.737	433.472	2608.110	4165.039	21.458
3	FIB-4	VCTE. Liver biopsy*	77.430	430.896	2590.950	4144.279	21.508
4	FIB-4		77.373	426.863	2566.925	4117.873	21.574
5	FIB-4. ELF	VCTE. Liver biopsy	77.462	433.619	2609.592	4166.221	21.453
6	FIB-4. ELF	VCTE. Liver biopsy*	77.394	432.080	2598.999	4153.794	21.483
7	FIB-4. ELF	Liver biopsy	77.510	433.487	2608.670	4165.433	21.455
8	FIB-4. ELF		77.373	430.595	2589.423	4142.669	21.512
9	FIB-4. VCTE	Liver biopsy	77.553	433.491	2608.598	4165.385	21.455
10	FIB-4. VCTE	Liver biopsy*	77.430	430.896	2590.950	4144.279	21.508
11	VCTE	Liver biopsy	77.789	433.399	2607.603	4164.625	21.458
12	VCTE	Liver biopsy*	77.529	428.367	2575.323	4126.841	21.552
13	No testing	No testing	77.373	435.467	2625.857	4182.262	21.424

* Indicates that liver biopsy was only given to patients with indeterminate VCTE.

Health care costs

A detailed breakdown of estimated costs is presented in Supplementary table 12 to 14. In Supplementary table 12 cost of primary care is defined as primary care visits, the cost of diagnostic algorithms is the cost of the algorithms, cost of other diagnostic test is diagnostic tests for HCC or DC outside of the surveillance program in specialist care, and cost of specialist care is defined as all cost in specialist care (further breakdown of cost in specialist care can be found in Supplementary table 13). In Supplementary table 13 the cost is categorized by health states with HCC cost being further broken down by HCC pre-treatment (the cost of HCC before receiving treatments, i.e., patients developing HCC outside of the surveillance program in specialist care and diagnosed via diagnostic algorithms), liver transplant, other curative treatment, and non-curative treatment (defined as the first year of treatment and subsequent years [post]). It should be noted that some of the transplant costs are not due to HCC, but to DC.

Supplementary table 14 summarizes the cost of diagnostic testing associated with each patient management strategy categorized in 10-year intervals. For transparency and understanding of model anatomy all cost were left undiscounted. Note that in all reported final results of total costs and cost-effectiveness, results are discounted accordingly.

Supplementary table 12. Breakdown of undiscounted mean costs

**Patient management strategies**			**Cost category**				
Strategy	Primary care testing	Specialist care testing	Primary care	Diagnostic algorithms	Other diagnostic tests	Specialist care	Total cost
1	FIB-4	VCTE. Liver biopsy	6044	2685	55	4712	13497
2	FIB-4	Liver biopsy	5842	3138	51	6196	15228
3	FIB-4	VCTE. Liver biopsy*	4415	1370	35	16018	21838
4	FIB-4		2007	177	17	34005	36207
5	FIB-4. ELF	VCTE. Liver biopsy	6123	1592	59	4186	11960
6	FIB-4. ELF	VCTE. Liver biopsy*	5326	976	45	9359	15706
7	FIB-4. ELF	Liver biopsy	6053	1750	56	4659	12518
8	FIB-4. ELF		4303	503	34	16800	21640
9	FIB-4. VCTE	Liver biopsy	6044	2240	55	4712	13052
10	FIB-4. VCTE	Liver biopsy*	4415	1033	35	16018	21501
11	VCTE	Liver biopsy	5886	6031	50	5826	17793
12	VCTE	Liver biopsy*	2788	1417	21	28109	32335
13	No testing	No testing	6529	0	110	2128	8767

FIB-4= Fibrosis 4 index, ELF=Enhanced Liver Fibrosis, VCTE= Vibration Controlled Transient Elastography, * indicates that liver biopsy was only given to patients with indeterminate VCTE.

Supplementary table 13. Breakdown of undiscounted mean costs in specialist care

**Patient management strategies**			**Cost category in specialist care**										
Strategy	Primary care testing	Specialist care testing	NAFLD-F2	F3	CC	DC	HCC pre treatment	LT	OCT	NCT	Post LT and OCT	Post NCT	Total cost
1	FIB-4	VCTE. Liver biopsy	638	382	1638	1334	48	227	62	92	193	98	4712
2	FIB-4	Liver biopsy	1938	446	1736	1322	52	237	69	94	200	100	6196
3	FIB-4	VCTE. Liver biopsy*	8665	2854	2114	1274	87	346	138	103	327	110	16018
4	FIB-4		24655	4001	2504	1225	134	513	232	113	509	120	34005
5	FIB-4. ELF	VCTE. Liver biopsy	268	334	1532	1348	45	224	59	91	189	96	4186
6	FIB-4. ELF	VCTE. Liver biopsy*	3052	2204	1881	1303	68	284	102	98	261	104	9359
7	FIB-4. ELF	Liver biopsy	607	376	1622	1336	48	227	62	92	192	98	4659
8	FIB-4. ELF		9217	3033	2140	1271	90	354	143	104	337	111	16800
9	FIB-4. VCTE	Liver biopsy	638	382	1638	1334	48	227	62	92	193	98	4712
10	FIB-4. VCTE	Liver biopsy*	8665	2854	2114	1274	87	346	138	103	327	110	16018
11	VCTE	Liver biopsy	1532	445	1784	1316	52	235	68	95	199	100	5826
12	VCTE	Liver biopsy*	19279	3711	2432	1235	119	457	201	111	448	117	28109
13	No testing	No testing	0	0	0	1540	20	215	41	70	169	74	2128

FIB-4= Fibrosis 4 index, ELF=Enhanced Liver Fibrosis, VCTE= Vibration Controlled Transient Elastography, * indicates that liver biopsy was only given to patients with indeterminate VCTE. HCC=hepatocellular carcinoma, DC=decompensated cirrhosis, surv= surveillance of HCC in specialist care, LT= liver transplant, OCT= other curative treatment for HCC (resection and ablation), NCT= not-curative treatment (Sorafenib, TACE and best supportive care)

Supplementary table 14. Undiscounted mean cost of diagnostic testing associated with the diagnostic algorithms

Patient management strategies			Cost of diagnostic algorithm testing					
Strategy	Primary care	Specialist care	First year	Year 3-10	Year 10-20	Year 20-30	Year 30-45	Total diagnostic testing
1	FIB-4	VCTE. Liver biopsy	472	746	1119	299	49	2 685
2	FIB-4	Liver biopsy	576	918	1379	257	8	3 138
3	FIB-4	VCTE. Liver biopsy*	305	406	491	138	30	1 370
4	FIB-4		70	64	38	5	1	177
5	FIB-4. ELF	VCTE. Liver biopsy	287	447	643	180	35	1 592
6	FIB-4. ELF	VCTE. Liver biopsy*	195	272	360	119	30	976
7	FIB-4. ELF	Liver biopsy	320	497	736	175	24	1 750
8	FIB-4. ELF		111	148	176	55	13	503
9	FIB-4. VCTE	Liver biopsy	402	633	941	230	33	2 240
10	FIB-4. VCTE	Liver biopsy*	231	310	374	99	20	1 033
11	VCTE	Liver biopsy	800	1279	3248	667	37	6 031
12	VCTE	Liver biopsy*	438	489	416	65	8	1 417
13	No testing	No testing	0	0	0	0	0	0

FIB-4= Fibrosis 4 index, ELF=Enhanced Liver Fibrosis, VCTE= Vibration Controlled Transient Elastography, * Indicates that liver biopsy was only given to patients with indeterminate VCTE.

Cost-effectiveness

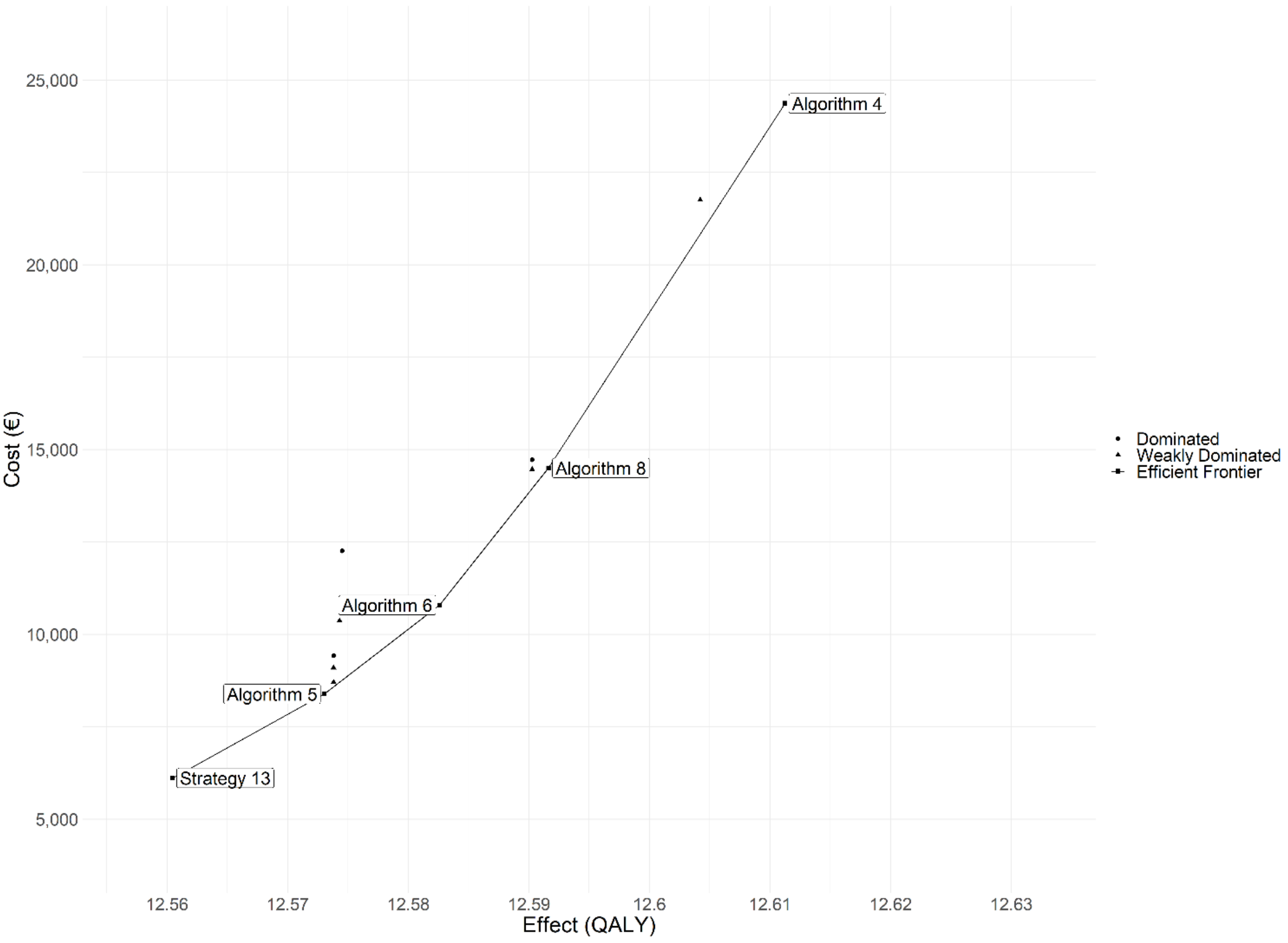

Supplementary figure 4. Cost-effectiveness plane with frontier

Sensitivity analysis

The procedure of conducted sensitivity analysis is described below. A summary of NHB for selected one-way analyses can be found in Supplementary table 15. Several sensitivity analyses were performed were Supplementary table 15 summarizes selected scenario analysis. In addition, did we perform more comprehensive sensitivity analysis regarding fibrosis progression, hypothetical treatment response and diagnostic test's characteristics.

QALY decrement

In the base case analysis, there was no QALY decrement associated with being under surveillance in specialist care. This assumption was tested in a sensitivity analysis by applying a QALY decrement (0.01) to all patients in specialist care, false positives and true positives, in order to capture the potential impact of anxiety and disutility of repeated doctor visits [29]. Previous studies have accounted for a one-time decrement of liver biopsy.[18*] The analysis indicated that a decrement in QALY had most impact on NHB of diagnostic algorithms with a larger number of patients residing in specialist care (see Supplementary table 15 for a summary and Supplementary table 17 for additional output). Still, the impact on the results was small, and the no-testing strategy still had the highest NHB, which was expected as a QALY-decrement lowers the value of testing.

Probability of HCC diagnosis outside the surveillance program

In the base case analysis, it was assumed that HCC patients could, with an annual probability, be diagnosed outside of the surveillance program. The importance of this assumption for the results was tested by setting the probability of being diagnosed with HCC to 0% for BCLC stages 0/A-C. For patients with stage D, it was assumed that the symptoms were so prominent that a probability of 0% was unrealistic and the base case probability of 100% was used. The impact on the overall cost-effectiveness result was small when implementing this assumption. What should be noted is that without allowing for HCC diagnosing outside of the surveillance programme almost no patient in the no-testing strategy received other curative treatment, see Supplementary table 15 for a summary and Supplementary table 18 for all outputs.

Time interval of retesting

In contrast to previous studies evaluating diagnostic test for patients with NAFLD, our model included reassessment of fibrosis stage in primary care. One crucial sensitivity analysis was therefore to evaluate to what extent the aspect of retesting impacted the result. Three scenario analyses were conducted, one with no retesting of patients, one with a 5-years interval, and one with a 10-year interval. The results indicated that no retesting had a positive impact on NHB of the diagnostic algorithms, although the no-testing strategy still had the highest NHB in all 3 scenarios (Supplementary table 15). This is in line with the base case result, as no-testing is preferred over any diagnostic algorithm a change in the algorithms which increase the similarities between no-testing and the algorithms were bound to increase NHB of testing strategies. See Supplementary table 19 to 21 for additional output.

Liver transplant

In the base case model, it was assumed that patients developing DC outside of specialist care (not previously known CC) had an equal probability of receiving liver transplant as patients with known CC developing DC. This was based on the assumption that once patients developed DC symptoms were so prominent that patients were identified with DC in the same cycle. It could be argued that once patients with not previously known CC were identified there was administrative guidelines needed to be followed which could affect the probability of liver transplant. We investigated the impact of this by setting the probability to 0% for the patients developing DC outside of specialist care. See Supplementary table 15 and 22. The analysis indicated that this assumption had a minor impact on the result. As expected, the NHB of no-testing decreases but was still higher than for any diagnostic algorithm.

Summary

Overall, the preciously described sensitivity analyses had no major impact on the result and no impact on the conclusion of the study.

Supplementary table 15. Summary of NHB in selected one-way sensitivity analyses

**Patient management strategies**			**Scenario tested**					
Strategy	Primary care	Specialist care	QALY decrement	Diagnosis of HCC outside of SC	No retesting	Retesting every 5th year	Retesting every 10th year	Liver transplant for patients with DC
1	FIB-4	VCTE. Liver biopsy	12.378 (-0.008)	12.381 (-0.005)	12.428 (0.042)	12.402 (0.017)	12.417 (0.031)	12.385 (-0.001)
2	FIB-4	Liver biopsy	12.357 (-0.010)	12.362 (-0.005)	12.425 (0.058)	12.390 (0.023)	12.411 (0.044)	12.366 (-0.001)
3	FIB-4	VCTE. Liver biopsy*	12.255 (-0.041)	12.293 (-0.003)	12.388 (0.092)	12.331 (0.035)	12.363 (0.067)	12.295 (-0.001)
4	FIB-4		12.027 (-0.097)	12.122 (-0.002)	12.277 (0.153)	12.170 (0.046)	12.227 (0.103)	12.124 (0.000)
5	FIB-4. ELF	VCTE. Liver biopsy	12.399 (-0.006)	12.401 (-0.005)	12.432 (0.026)	12.415 (0.010)	12.424 (0.019)	12.405 (-0.001)
6	FIB-4. ELF	VCTE. Liver biopsy*	12.345 (-0.022)	12.363 (-0.004)	12.414 (0.048)	12.384 (0.018)	12.401 (0.034)	12.366 (-0.001)
7	FIB-4. ELF	Liver biopsy	12.392 (-0.007)	12.395 (-0.005)	12.431 (0.031)	12.411 (0.012)	12.422 (0.023)	12.399 (-0.001)
8	FIB-4. ELF		12.258 (-0.043)	12.298 (-0.003)	12.387 (0.086)	12.334. (0.032)	12.364 (0.062)	12.301 (0.000)
9	FIB-4. VCTE	Liver biopsy	12.284 (-0.008)	12.387 (-0.005)	12.429 (0.037)	12.406 (0.014)	12.419 (0.027)	12.391 (-0.001)
10	FIB-4. VCTE	Liver biopsy*	12.260 (-0.041)	12.298 (-0.003)	12.389 (0.088)	12.335 (0.034)	12.365 (0.064)	12.300 (-0.001)
11	VCTE	Liver biopsy	12.320 (-0.010)	12.298 (-0.005)	12.421 (0.092)	12.366 (0.037)	12.395 (0.066)	12.329 (-0.001)
12	VCTE	Liver biopsy*	12.092 (-0.077)	12.167 (-0.002)	12.321 (0.151)	12.221 (0.051)	12.276 (0.107)	12.160 (0.000)
13	No testing	No testing	12.437 (-0.001)	12.433 (-0.005)	12.438 (0.000)	12.438 (0.000)	12.438 (0.000)	12.438 (-0.001)

FIB-4= Fibrosis 4 index, ELF=Enhanced Liver Fibrosis, VCTE= Vibration Controlled Transient Elastography * indicates that liver biopsy was only given to patients with indeterminate VCTE. Number inside parentheses indicate changes in NHB from base case result.

Progression

A sensitivity analysis of a more rapid disease progression for fibrosis stages was performed in order to test whether the result was sensitive to fast progressors. The reasoning behind the analysis was that if it was possible to stratify the patient population and identify patients with rapid disease progression, diagnostic algorithms might be more valuable (and cost-effective) for those patients. However, the results indicated that a faster disease progression only had limited effect on the result and the conclusion of the result did not change. Hence, in current care risk stratifying test to identify rapid disease progresses is not cost-effective, see Supplementary table 23.

Treatment response

Lifetime interventions resulting in weight loss are hard to maintain over time.[47*] Though studies have indicated the importance of weight loss and lifestyle intervention in the management of NAFLD[30] the effect on histological liver fibrosis status has not been established and requires further scrutiny.[48*] As evidence on treatment and intervention for patients with NAFLD is associated with uncertainty we performed a sensitivity analysis where a range of treatment response scenarios were tested. Treatment response was applied as a hazard ratio to slow down disease progression through regression or progression. Treatment response was applied to patients with known NAFLD, NASH or fibrosis status in primary care. Hence patient in the no-testing strategy was not subjected to treatment. Note that the

probability to develop DC was not affected by added treatment response, rather the treatment *only* affected the probability to transition between previously mentioned health states. The sensitivity analysis was performed as three different scenarios; treatment response was applied to early disease stages (NAFLD-F1), to late fibrosis stages (F2-CC), and to all fibrosis stages (NAFLD-CC). For these three scenarios we applied a range of treatment response magnitudes to fully investigate the impact of a potential treatment response.

The result indicates that, with even a modest treatment response (HR 1.1) applied to fibrosis regression, NAFLD-CC, several diagnostic algorithms yielded higher NHB than no-testing, Supplementary table 24. Algorithm number 5 yielded the highest NHB in all scenarios investigating treatment response. Similar result was yielded when treatment response was applied to the probability to progress between health states (HR<1), see Supplementary table 24. When the treatment response was applied only to NAFLD-F1 a slightly higher response (HR 1.2) was needed for testing to yield higher NHB than no-testing, see Supplementary table 25. For patients with F2-CC a modest treatment response (HR 1.1) was enough for the diagnostic algorithms to yield higher NHB compared with no testing, see Supplementary table 26. It should be noted that there are several simplifications associated with these analyses of treatment response and that a more extensive analysis of implications of different treatment characteristic may be appropriate to fully incorporate the effect of treatments. For example, treatment was applied equally to all health states, and it could be discussed if behavioural changes or pharmacological treatments has varying effect due to severity of disease. In addition, did we not take into account how treatment response could have different characteristics, as discussed in previous literature.[6] Nevertheless, these analyses indicate how our model behaves if treatments should become available.

Diagnostic tests' characteristics

A sensitivity analysis was undertaken to evaluate how different cut-offs affected the result of the study. The cut-offs were set to yield sensitivity or specificity at 90% in the three non-invasive diagnostic tests, see Supplementary table 16. The result indicates that improvements in sensitivity with a lower cut-off and therefore a lower specificity, had a negative impact on NHB in the diagnostic algorithms, see Supplementary figure 27. At the opposite, improvements in specificity, at the cost of a lower sensitivity (higher cut-off) increased NHB of the diagnostic algorithms. However, no-testing still yielded higher NHB. In other words, in current care diagnostic algorithms with improved specificity to correctly classify patient without the disease is to be preferred as current care is not sufficiently effective when it comes to improve the prognosis of true positives to balance the cost of testing.

Supplementary table 16. Diagnostic tests' characteristics

Diagnostic	Cut-off	Sensitivity	Specificity	Source
FIB-4				
NAFLD-F3	0.880	0.900	0.390	[31*]
NAFLD-F3	2.310	0.380	0.900	[31*]
CC	0.880	0.950		Assumption
CC	2.310	0.750		Assumption
ELF				
NAFLD-F3	8.100	0.900	0.470	[33*]
NAFLD-F3	10.180	0.570	0.900	[33*]
CC	8.100	0.950		Assumption
CC	10.180	0.750		Assumption
VCTE				
NAFLD-F3	7.400	0.900	0.600	[31*]
NAFLD-F3	12.100	0.550	0.900	[31*]
CC	7.400	0.950		Assumption
CC	12.100	0.750		Assumption

FIB-4= Fibrosis 4 index, ELF=Enhanced Liver Fibrosis, VCTE= Vibration Controlled Transient Elastography

Supplementary table 17. One-way sensitivity analysis with a QALY decrement of 0.01 in specialist care

**Patient management strategies**			**Cost-effectiveness outcomes**	
Strategy	Primary care	Specialist care	QALY	NHB
1	FIB-4	VCTE. Liver biopsy	12.566	12.378
2	FIB-4	Liver biopsy	12.564	12.357
3	FIB-4	VCTE. Liver biopsy*	12.549	12.255
4	FIB-4		12.514	12.027
5	FIB-4. ELF	VCTE. Liver biopsy	12.567	12.399
6	FIB-4. ELF	VCTE. Liver biopsy*	12.561	12.345
7	FIB-4. ELF	Liver biopsy	12.566	12.392
8	FIB-4. ELF		12.548	12.258
9	FIB-4. VCTE	Liver biopsy	12.566	12.384
10	FIB-4. VCTE	Liver biopsy*	12.549	12.260
11	VCTE	Liver biopsy	12.565	12.320
12	VCTE	Liver biopsy*	12.527	12.092
13	No testing	No testing	12.559	12.437

FIB-4= Fibrosis 4 index, ELF=Enhanced Liver Fibrosis, VCTE= Vibration Controlled Transient Elastography, * indicates that liver biopsy was only given to patients with indeterminate VCTE.

Supplementary table 18. One-way sensitivity analysis of no probability of diagnosis of HCC outside of surveillance program in BCLC stage 0/A-C

**Patient management strategies**			**Clinical outcomes (cases per 10 000)**								**Cost-effectiveness outcomes (per patient mean)**			
Strategy	Primary care	Specialist care	Number of DC	Number of HCC	Number of detected HCC (surv)	Number of detected HCC (not surv)	Number of LT	Number of OCT	Number of NCT	10-year mortality	QALY	Cost (€)	Life years	NHB
1	FIB-4	VCTE. Liver biopsy	447.739	668.568	106.717	5.374	23.350	24.193	78.544	1362.743	12.567	9 289	15.055	12.381
2	FIB-4	Liver biopsy	443.579	668.416	119.631	6.014	24.908	31.425	82.261	1362.336	12.567	10 232	15.056	12.362
3	FIB-4	VCTE. Liver biopsy*	427.495	667.975	234.395	4.728	40.901	107.779	102.578	1350.751	12.585	14 628	15.088	12.293
4	FIB-4		411.080	667.567	387.883	4.413	65.251	211.222	125.647	1332.887	12.609	24 323	15.130	12.122
5	FIB-4. ELF	VCTE. Liver biopsy	452.191	668.688	99.925	5.242	22.806	20.967	75.799	1363.480	12.566	8 250	15.053	12.401
6	FIB-4. ELF	VCTE. Liver biopsy*	437.340	668.250	172.990	4.460	31.916	67.820	91.338	1356.285	12.576	10 677	15.072	12.363
7	FIB-4. ELF	Liver biopsy	448.416	668.589	105.891	5.367	23.297	23.825	78.176	1362.801	12.567	8 569	15.055	12.395
8	FIB-4. ELF		426.381	667.957	243.176	4.668	42.151	113.699	104.037	1349.538	12.587	14 409	15.090	12.298
9	FIB-4. VCTE	Liver biopsy	447.739	668.568	106.717	5.374	23.350	24.193	78.544	1362.743	12.567	8 962	15.055	12.387
10	FIB-4. VCTE	Liver biopsy*	427.495	667.975	234.395	4.728	40.901	107.779	102.578	1350.751	12.585	14 369	15.088	12.298
11	VCTE	Liver biopsy	441.660	668.384	118.376	5.775	24.494	30.173	82.653	1361.937	12.567	12 128	15.056	12.325
12	VCTE	Liver biopsy*	414.192	667.657	339.511	4.817	57.160	177.773	119.552	1339.277	12.601	21 692	15.116	12.167
13	No testing	No testing	516.696	701.399	40.988	6.327	21.020	0.127	42.710	1373.018	12.552	5 954	15.034	12.433

NHB calculated with a threshold of €50 000 FIB-4= Fibrosis 4 index, ELF=Enhanced Liver Fibrosis, VCTE= Vibration Controlled Transient Elastography, * indicates that liver biopsy was only given to patients with indeterminate VCTE. DC=decompensated cirrhosis, surv= surveillance program of HCC in specialist care, not surv= patients opportunistically detected with HCC outside of surveillance program. LT= liver transplant, OCT= other curative treatment for HCC (resection and ablation), NCT= not-curative treatment (Sorafenib, TACE and best supportive care).

Supplementary table 19. One-way sensitivity analysis of no retesting

Patient management strategies			Clinical outcomes (cases per 10 000)								Cost-effectiveness outcomes (per patient mean)			
Strategy	Primary care	Specialist care	Number of DC	Number of HCC	Number of detected HCC (surv)	Number of detected HCC (not surv)	Number of LT	Number of OCT	Number of NCT	10-year mortality	QALY	Cost (€)	Life years	NHB
1	FIB-4	VCTE. Liver biopsy	500.957	670.502	56.974	88.936	28.576	43.948	89.423	1357.690	12.566	6 924	15.058	12.428
2	FIB-4	Liver biopsy	499.504	670.499	58.443	88.644	28.614	44.417	90.046	1357.197	12.567	7 058	15.059	12.425
3	FIB-4	VCTE. Liver biopsy*	481.590	670.097	115.744	77.213	34.732	75.285	98.356	1348.183	12.576	9 430	15.075	12.388
4	FIB-4		456.125	669.389	225.068	55.412	48.384	135.992	110.706	1333.626	12.593	15 771	15.103	12.277
5	FIB-4. ELF	VCTE. Liver biopsy	502.534	670.498	55.375	89.253	28.534	43.437	88.745	1358.323	12.566	6 707	15.058	12.432
6	FIB-4. ELF	VCTE. Liver biopsy*	490.254	670.269	87.835	82.778	31.616	60.370	94.320	1352.606	12.572	7 866	15.067	12.414
7	FIB-4. ELF	Liver biopsy	501.230	670.503	56.698	88.991	28.569	43.860	89.306	1357.775	12.566	6 768	15.058	12.431
8	FIB-4. ELF		479.586	670.053	121.959	75.973	35.377	78.625	99.282	1347.177	12.577	9 509	15.077	12.387
9	FIB-4. VCTE	Liver biopsy	500.957	670.502	56.974	88.936	28.576	43.948	89.423	1357.690	12.566	6 855	15.058	12.429
10	FIB-4. VCTE	Liver biopsy*	481.590	670.097	115.744	77.213	34.732	75.285	98.356	1348.183	12.576	9 358	15.075	12.389
11	VCTE	Liver biopsy	498.597	670.501	59.362	88.462	28.638	44.711	90.436	1356.833	12.567	7 299	15.059	12.421
12	VCTE	Liver biopsy*	463.928	669.677	181.658	64.070	42.581	111.254	106.745	1338.705	12.587	13 301	15.092	12.321
13	No testing	No testing	516.696	701.399	40.988	73.960	28.155	38.837	82.634	1364.660	12.560	6 111	15.050	12.438

NHB calculated with a threshold of €50 000 FIB-4= Fibrosis 4 index, ELF=Enhanced Liver Fibrosis, VCTE= Vibration Controlled Transient Elastography, * indicates that liver biopsy was only given to patients with indeterminate VCTE. DC=decompensated cirrhosis, surv= surveillance program of HCC in specialist care, not surv= patients opportunistically detected with HCC outside of surveillance program. LT= liver transplant, OCT= other curative treatment for HCC (resection and ablation), NCT= not-curative treatment (Sorafenib, TACE and best supportive care).

Supplementary table 20. One-way sensitivity analysis of retesting in 5-year intervals

**Patient management strategies**			**Clinical outcomes (cases per 10 000)**								**Cost-effectiveness outcomes (per patient mean)**			
Strategy	Primary care	Specialist care	Number of DC	Number of HCC	Number of detected HCC (surv)	Number of detected HCC (not surv)	Number of LT	Number of OCT	Number of NCT	10-year mortality	QALY	Cost (€)	Life years	NHB
1	FIB-4	VCTE. Liver biopsy	461.752	668.952	92.650	81.567	29.339	55.054	104.425	1356.849	12.571	8 462	15.065	12.402
2	FIB-4	Liver biopsy	457.538	668.811	102.718	80.063	30.297	59.694	106.682	1356.435	12.572	9 113	15.066	12.390
3	FIB-4	VCTE. Liver biopsy*	440.621	668.347	196.218	61.506	41.398	112.894	116.702	1347.120	12.585	12 725	15.089	12.331
4	FIB-4		419.815	667.772	345.192	33.258	61.839	198.658	129.134	1332.366	12.605	21 751	15.125	12.170
5	FIB-4. ELF	VCTE. Liver biopsy	466.215	669.071	86.882	82.613	29.029	52.862	102.492	1357.488	12.570	7 763	15.064	12.415
6	FIB-4. ELF	VCTE. Liver biopsy*	451.403	668.650	145.645	70.988	35.174	84.967	110.752	1351.638	12.579	9 702	15.078	12.384
7	FIB-4. ELF	Liver biopsy	462.458	668.972	91.874	81.714	29.307	54.776	104.140	1356.913	12.571	7 984	15.065	12.411
8	FIB-4. ELF		439.155	668.316	204.714	59.848	42.410	117.757	117.586	1346.087	12.586	12 633	15.091	12.334
9	FIB-4. VCTE	Liver biopsy	461.752	668.952	92.650	81.567	29.339	55.054	104.425	1356.849	12.571	8 246	15.065	12.406
10	FIB-4. VCTE	Liver biopsy*	440.621	668.347	196.218	61.506	41.398	112.894	116.702	1347.120	12.585	12 534	15.089	12.335
11	VCTE	Liver biopsy	455.408	668.771	102.514	79.916	30.031	59.113	107.304	1356.073	12.572	10 307	15.067	12.366
12	VCTE	Liver biopsy*	424.311	667.928	293.338	43.232	54.250	167.948	126.026	1337.562	12.598	18 882	15.112	12.221
13	No testing	No testing	516.696	701.399	40.988	73.960	28.155	38.837	82.634	1364.660	12.560	6 111	15.050	12.438

NHB calculated with a threshold of €50 000 FIB-4= Fibrosis 4 index, ELF=Enhanced Liver Fibrosis, VCTE= Vibration Controlled Transient Elastography, * indicates that liver biopsy was only given to patients with indeterminate VCTE. DC=decompensated cirrhosis, surv= surveillance program of HCC in specialist care, not surv= patients opportunistically detected with HCC outside of surveillance program. LT= liver transplant, OCT= other curative treatment for HCC (resection and ablation), NCT= not-curative treatment (Sorafenib, TACE and best supportive care).

Supplementary table 21. One-way sensitivity analysis of retesting in 10-year intervals

**Patient management strategies**			**Clinical outcomes (cases per 10 000)**								**Cost-effectiveness outcomes (per patient mean)**			
Strategy	Primary care	Specialist care	Number of DC	Number of HCC	Number of detected HCC (surv)	Number of detected HCC (not surv)	Number of LT	Number of OCT	Number of NCT	10-year mortality	QALY	Cost (€)	Life years	NHB
1	FIB-4	VCTE. Liver biopsy	481.413	669.562	73.658	85.399	28.804	48.767	96.840	1357.690	12.568	7 568	15.061	12.417
2	FIB-4	Liver biopsy	478.290	669.472	78.126	84.679	29.073	50.474	98.301	1357.197	12.568	7 870	15.061	12.411
3	FIB-4	VCTE. Liver biopsy*	460.033	668.993	154.536	69.535	37.680	92.786	107.947	1348.183	12.580	10 838	15.080	12.363
4	FIB-4		435.577	668.290	287.539	43.921	55.065	167.871	121.161	1333.626	12.598	18 567	15.112	12.227
5	FIB-4. ELF	VCTE. Liver biopsy	484.783	669.647	70.178	86.073	28.695	47.630	95.433	1358.323	12.567	7 156	15.060	12.424
6	FIB-4. ELF	VCTE. Liver biopsy*	470.563	669.280	115.569	77.044	33.183	71.783	102.628	1352.606	12.574	8 656	15.071	12.401
7	FIB-4. ELF	Liver biopsy	481.986	669.579	73.106	85.509	28.790	48.592	96.609	1357.775	12.568	7 275	15.060	12.422
8	FIB-4. ELF		458.172	668.951	162.296	68.005	38.540	97.089	108.937	1347.177	12.581	10 847	15.082	12.364
9	FIB-4. VCTE	Liver biopsy	481.413	669.562	73.658	85.399	28.804	48.767	96.840	1357.690	12.568	7 436	15.061	12.419
10	FIB-4. VCTE	Liver biopsy*	460.033	668.993	154.536	69.535	37.680	92.786	107.947	1348.183	12.580	10 712	15.080	12.365
11	VCTE	Liver biopsy	476.433	669.429	79.243	84.375	29.027	50.684	98.967	1356.833	12.569	8 685	15.062	12.395
12	VCTE	Liver biopsy*	441.948	668.520	236.842	53.724	47.973	138.398	117.331	1338.705	12.591	15 777	15.100	12.276
13	No testing	No testing	516.696	701.399	40.988	73.960	28.155	38.837	82.634	1364.660	12.560	6 111	15.050	12.438

NHB calculated with a threshold of €50 000 FIB-4= Fibrosis 4 index, ELF=Enhanced Liver Fibrosis, VCTE= Vibration Controlled Transient Elastography, * indicates that liver biopsy was only given to patients with indeterminate VCTE. DC=decompensated cirrhosis, surv= surveillance program of HCC in specialist care, not surv= patients opportunistically detected with HCC outside of surveillance program. LT= liver transplant, OCT= other curative treatment for HCC (resection and ablation), NCT= not-curative treatment (Sorafenib, TACE and best supportive care).

Supplementary table 22. One-way sensitivity analysis with a probability of liver transplant set to 0% for patients with DC not monitored in specialist care

**Patient management strategies**			**Clinical outcomes (cases per 10 000)**								**Cost-effectiveness outcomes (per patient mean)**			
Strategy	Primary care	Specialist care	Number of DC	Number of HCC	Number of detected HCC (surv)	Number of detected HCC (not surv)	Number of LT	Number of OCT	Number of NCT	10-year mortality	QALY	Cost (€)	Life years	NHB
1	FIB-4	VCTE. Liver biopsy	447.739	669.388	106.424	78.944	22.545	59.584	110.034	1355.497	12.572	9 376	15.066	12.385
2	FIB-4	Liver biopsy	443.579	669.174	119.086	77.117	24.460	65.796	112.372	1355.103	12.573	10 319	15.068	12.366
3	FIB-4	VCTE. Liver biopsy*	427.495	668.490	233.598	54.526	40.742	131.951	123.229	1344.848	12.589	14 692	15.096	12.295
4	FIB-4		411.080	667.832	386.932	25.550	64.807	221.642	133.968	1329.100	12.611	24 350	15.134	12.124
5	FIB-4. ELF	VCTE. Liver biopsy	452.191	669.575	99.767	80.092	21.493	56.811	108.122	1356.218	12.571	8 336	15.065	12.405
6	FIB-4. ELF	VCTE. Liver biopsy*	437.340	668.914	172.453	65.758	31.422	97.353	117.247	1349.803	12.581	10 752	15.082	12.366
7	FIB-4. ELF	Liver biopsy	448.416	669.419	105.611	79.093	22.410	59.267	109.770	1355.553	12.572	8 657	15.066	12.399
8	FIB-4. ELF		426.381	668.454	242.375	52.831	42.000	137.085	123.985	1343.769	12.591	14 471	15.098	12.301
9	FIB-4. VCTE	Liver biopsy	447.739	669.388	106.424	78.944	22.545	59.584	110.034	1355.497	12.572	9 050	15.066	12.391
10	FIB-4. VCTE	Liver biopsy*	427.495	668.490	233.598	54.526	40.742	131.951	123.229	1344.848	12.589	14 433	15.096	12.300
11	VCTE	Liver biopsy	441.660	669.111	117.869	77.126	24.343	64.727	112.802	1354.712	12.573	12 216	15.068	12.329
12	VCTE	Liver biopsy*	414.192	667.968	338.518	34.953	57.102	192.618	131.579	1334.664	12.603	21 732	15.121	12.169
13	No testing	No testing	516.696	703.271	42.333	73.960	11.748	38.837	83.844	1365.550	12.557	6 007	15.044	12.437

NHB calculated with a threshold of €50 000 FIB-4= Fibrosis 4 index, ELF=Enhanced Liver Fibrosis, VCTE= Vibration Controlled Transient Elastography, * indicates that liver biopsy was only given to patients with indeterminate VCTE. DC=decompensated cirrhosis, surv= surveillance program of HCC in specialist care, not surv= patients opportunistically detected with HCC outside of surveillance program. LT= liver transplant, OCT= other curative treatment for HCC (resection and ablation), NCT= not-curative treatment (Sorafenib, TACE and best supportive care).

Supplementary table 23. One-way sensitivity analysis of faster fibrosis progression reported as NHB

Patient management strategies			Scenarios tested							
Strategy	Primary care	Specialist care	HR 1.5	HR 2	HR 2.5	HR 3	HR 3.5	HR 4	HR 4.5	HR 5
1	FIB-4	VCTE. Liver biopsy	11.673	11.020	10.427	9.891	9.407	8.969	8.572	8.213
2	FIB-4	Liver biopsy	11.659	11.010	10.419	9.885	9.402	8.965	8.570	8.211
3	FIB-4	VCTE. Liver biopsy*	11.595	10.953	10.372	9.846	9.370	8.940	8.551	8.198
4	FIB-4		11.444	10.822	10.259	9.749	9.287	8.870	8.492	8.148
5	FIB-4. ELF	VCTE. Liver biopsy	11.690	11.036	10.441	9.904	9.418	8.978	8.581	8.221
6	FIB-4. ELF	VCTE. Liver biopsy*	11.656	11.006	10.416	9.884	9.402	8.967	8.574	8.217
7	FIB-4. ELF	Liver biopsy	11.686	11.032	10.439	9.902	9.416	8.978	8.581	8.221
8	FIB-4. ELF		11.601	10.959	10.378	9.852	9.377	8.947	8.558	8.205
9	FIB-4. VCTE	Liver biopsy	11.679	11.026	10.433	9.897	9.412	8.973	8.577	8.217
10	FIB-4. VCTE	Liver biopsy*	11.600	10.958	10.376	9.850	9.374	8.944	8.555	8.202
11	VCTE	Liver biopsy	11.625	10.980	10.393	9.862	9.381	8.947	8.553	8.197
12	VCTE	Liver biopsy*	11.484	10.857	10.289	9.774	9.309	8.888	8.507	8.160
13	No testing	No testing	11.721	11.062	10.464	9.922	9.432	8.990	8.590	8.227

NHB calculated with a threshold of €50 000 FIB-4= Fibrosis 4 index, ELF=Enhanced Liver Fibrosis, VCTE= Vibration Controlled Transient Elastography, * indicates that liver biopsy was only given to patients with indeterminate VCTE. , HR=Hazard ratios.

Supplementary table 24. One-way sensitivity analysis of treatment response in NAFLD-CC reported as NHB

**Patient management strategies**			**Treatment response on regression**					**Treatment response on progression**				
Strategy	Primary care	Specialist care	HR 1.1	HR 1.2	HR 1.3	HR 1.4	HR 1.5	HR 0.9	HR 0.8	HR 0.7	HR 0.6	HR 0.5
1	FIB-4	VCTE. Liver biopsy	12.453	12.519	12.583	12.646	12.707	12.527	12.671	12.817	12.966	13.117
2	FIB-4	Liver biopsy	12.434	12.499	12.562	12.624	12.684	12.506	12.648	12.791	12.938	13.087
3	FIB-4	VCTE. Liver biopsy*	12.345	12.394	12.442	12.489	12.535	12.407	12.521	12.631	12.754	12.874
4	FIB-4		12.151	12.177	12.204	12.229	12.255	12.183	12.243	12.281	12.363	12.424
5	FIB-4. ELF	VCTE. Liver biopsy	*12.474*	*12.540*	*12.605*	*12.668*	*12.730*	*12.547*	*12.692*	*12.840*	*12.990*	*13.142*
6	FIB-4. ELF	VCTE. Liver biopsy*	12.425	12.481	12.537	12.592	12.645	12.496	12.627	12.760	12.898	13.037
7	FIB-4. ELF	Liver biopsy	12.468	12.534	12.598	12.661	12.722	12.541	12.685	12.832	12.982	13.133
8	FIB-4. ELF		12.350	12.397	12.444	12.489	12.534	12.411	12.523	12.630	12.752	12.869
9	FIB-4. VCTE	Liver biopsy	12.460	12.526	12.590	12.653	12.714	12.533	12.677	12.824	12.973	13.124
10	FIB-4. VCTE	Liver biopsy*	12.351	12.399	12.447	12.494	12.540	12.413	12.527	12.636	12.760	12.879
11	VCTE	Liver biopsy	12.396	12.461	12.524	12.585	12.646	12.468	12.609	12.752	12.899	13.047
12	VCTE	Liver biopsy*	12.204	12.238	12.271	12.304	12.336	12.247	12.325	12.388	12.485	12.565
13	No testing	No testing	12.438	12.438	12.438	12.438	12.438	12.438	12.438	12.399	12.438	12.438

NHB calculated with a threshold of €50 000 FIB-4= Fibrosis 4 index, ELF=Enhanced Liver Fibrosis, VCTE= Vibration Controlled Transient Elastography, * indicates that liver biopsy was only given to patients with indeterminate VCTE, HR=Hazard ratios.

Supplementary table 25. One-way sensitivity analysis of treatment response in NAFLD-F1 reported as NHB

**Patient management strategies**			**Treatment response on regression**					**Treatment response on progression**				
Strategy	Primary care	Specialist care	HR 1.1	HR 1.2	HR 1.3	HR 1.4	HR 1.5	HR 0.9	HR 0.8	HR 0.7	HR 0.6	HR 0.5
1	FIB-4	VCTE. Liver biopsy	12.407	12.429	12.450	12.471	12.491	12.480	12.580	12.683	12.791	12.902
2	FIB-4	Liver biopsy	12.389	12.410	12.431	12.451	12.471	12.460	12.558	12.659	12.764	12.874
3	FIB-4	VCTE. Liver biopsy*	12.314	12.333	12.351	12.368	12.385	12.376	12.459	12.545	12.634	12.727
4	FIB-4		12.134	12.144	12.154	12.164	12.174	12.166	12.208	12.252	12.297	12.343
5	FIB-4. ELF	VCTE. Liver biopsy	12.428	*12.449*	*12.470*	*12.491*	*12.511*	*12.501*	*12.601*	*12.705*	*12.813*	*12.926*
6	FIB-4. ELF	VCTE. Liver biopsy*	12.388	12.409	12.429	12.448	12.468	12.458	12.554	12.653	12.757	12.865
7	FIB-4. ELF	Liver biopsy	12.422	12.443	12.465	12.485	12.505	12.495	12.594	12.698	12.806	12.918
8	FIB-4. ELF		12.320	12.338	12.356	12.373	12.390	12.381	12.462	12.547	12.636	12.727
9	FIB-4. VCTE	Liver biopsy	12.414	12.436	12.457	12.477	12.497	12.487	12.586	12.690	12.797	12.909
10	FIB-4. VCTE	Liver biopsy*	12.320	12.338	12.356	12.373	12.390	12.381	12.464	12.550	12.639	12.732
11	VCTE	Liver biopsy	12.351	12.373	12.394	12.414	12.434	12.423	12.520	12.621	12.726	12.836
12	VCTE	Liver biopsy*	12.183	12.196	12.209	12.221	12.234	12.225	12.282	12.341	12.401	12.464
13	No testing	No testing	*12.438*	12.438	12.438	12.438	12.438	12.438	12.438	12.438	12.438	12.438

NHB calculated with a threshold of €50 000 FIB-4= Fibrosis 4 index, ELF=Enhanced Liver Fibrosis, VCTE= Vibration Controlled Transient Elastography, * indicates that liver biopsy was only given to patients with indeterminate VCTE, HR=Hazard ratios.

Supplementary table 26. One-way sensitivity analysis of treatment response in F2-CC reported as NHB

**Patient management strategies**			**Treatment response on regression**					**Treatment response on progression**				
Strategy	Primary care	Specialist care	HR 1.1	HR 1.2	HR 1.3	HR 1.4	HR 1.5	HR 0.9	HR 0.8	HR 0.7	HR 0.6	HR 0.5
1	FIB-4	VCTE. Liver biopsy	12.431	12.475	12.517	12.558	12.597	12.433	12.494	12.556	12.619	12.684
2	FIB-4	Liver biopsy	12.412	12.455	12.496	12.537	12.576	12.414	12.474	12.536	12.598	12.662
3	FIB-4	VCTE. Liver biopsy*	12.327	12.357	12.386	12.415	12.442	12.328	12.373	12.417	12.463	12.510
4	FIB-4		12.140	12.157	12.173	12.188	12.204	12.142	12.165	12.189	12.213	12.238
5	FIB-4. ELF	VCTE. Liver biopsy	*12.451*	*12.495*	*12.538*	*12.580*	*12.620*	*12.453*	*12.514*	*12.577*	*12.640*	*12.705*
6	FIB-4. ELF	VCTE. Liver biopsy*	12.403	12.439	12.474	12.507	12.540	12.405	12.456	12.509	12.563	12.618
7	FIB-4. ELF	Liver biopsy	12.445	12.489	12.532	12.573	12.612	12.447	12.508	12.571	12.634	12.699
8	FIB-4. ELF		12.331	12.360	12.388	12.416	12.443	12.333	12.376	12.420	12.464	12.510
9	FIB-4. VCTE	Liver biopsy	12.437	12.481	12.523	12.564	12.604	12.439	12.500	12.562	12.626	12.691
10	FIB-4. VCTE	Liver biopsy*	12.332	12.362	12.391	12.420	12.448	12.334	12.378	12.423	12.469	12.516
11	VCTE	Liver biopsy	12.374	12.417	12.458	12.498	12.537	12.376	12.436	12.497	12.559	12.623
12	VCTE	Liver biopsy*	12.190	12.211	12.231	12.250	12.270	12.192	12.222	12.253	12.284	12.316
13	No testing	No testing	12.438	12.438	12.438	12.438	12.438	12.438	12.438	12.438	12.438	12.438

NHB calculated with a threshold of €50 000 FIB-4= Fibrosis 4 index, ELF=Enhanced Liver Fibrosis, VCTE= Vibration Controlled Transient Elastography, * indicates that liver biopsy was only given to patients with indeterminate VCTE, HR=Hazard ratios.

Supplementary table 27. One-way sensitivity analysis of diagnostic testing reported as NHB

**Patient management strategies**			**Sensitivity 90%**				**Specificity 90%**			
			FIB-4	ELF	VCTE	All**	FIB-4	ELF	VCTE	All**
Strategy	Primary care	Specialist care	SP 39%	SP 47%	SP 60%		SE 38%	SE 57%	SE 55%	
1	FIB-4	VCTE. Liver biopsy	12.358 (-0.028)	12.385 (0.000)	12.377 (-0.008)	12.342 (-0.043)	12.408 (0.022)	12.385 (0.000)	12.393 (0.008)	12.411 (0.026)
2	FIB-4	Liver biopsy	12.324 (-0.043)	12.367 (0.000)	12.368 (0.001)	12.324 (-0.043)	12.401 (0.034)	12.367 (0.000)	12.367 (0.000)	12.401 (0.034)
3	FIB-4	VCTE. Liver biopsy*	12.222 (-0.074)	12.296 (0.000)	12.248 (-0.048)	12.154 (-0.141)	12.365 (0.070)	12.296 (0.000)	12.339 (0.043)	12.385 (0.089)
4	FIB-4		12.024 (-0.100)	12.124 (0.000)	12.124 (0.000)	12.024 (-0.100)	12.271 (0.147)	12.124 (0.000)	12.124 (0.000)	12.271 (0.147)
5	FIB-4. ELF	VCTE. Liver biopsy	12.395 (-0.010)	12.396 (-0.009)	12.403 (-0.002)	12.369 (-0.036)	12.415 (0.010)	12.411 (0.005)	12.409 (0.004)	12.419 (0.014)
6	FIB-4. ELF	VCTE. Liver biopsy*	12.340 (-0.027)	12.333 (-0.033)	12.352 (-0.015)	12.231 (-0.136)	12.395 (0.028)	12.383 (0.016)	12.383 (0.017)	12.409 (0.042)
7	FIB-4. ELF	Liver biopsy	12.385 (-0.015)	12.383 (-0.016)	12.400 (0.000)	12.353 (-0.046)	12.413 (0.013)	12.408 (0.008)	12.400 (0.000)	12.417 (0.017)
8	FIB-4. ELF		12.233 (-0.068)	12.207 (-0.094)	12.302 (0.000)	12.108 (-0.194)	12.367 (0.066)	12.344 (0.043)	12.302 (0.000)	12.386 (0.085)
9	FIB-4. VCTE	Liver biopsy	12.369 (-0.022)	12.392 (0.000)	12.384 (-0.008)	12.354 (-0.038)	12.410 (0.019)	12.392 (0.000)	12.400 (0.008)	12.414 (0.022)
10	FIB-4. VCTE	Liver biopsy*	12.230 (-0.071)	12.302 (0.000)	12.252 (-0.049)	12.160 (-0.141)	12.368 (0.067)	12.302 (0.000)	12.346 (0.045)	12.388 (0.087)
11	VCTE	Liver biopsy	12.329 (0.000)	12.329 (0.000)	12.308 (-0.021)	12.308 (-0.021)	12.329 (0.000)	12.329 (0.000)	12.352 (0.022)	12.352 (0.022)
12	VCTE	Liver biopsy*	12.169 (0.000)	12.169 (0.000)	12.091 (-0.078)	12.091 (-0.078)	12.169 (0.000)	12.169 (0.000)	12.256 (0.087)	12.256 (0.087)
13	No testing	No testing	12.438 (0.000)	12.438 (0.000)	12.438 (0.000)	12.438 (0.000)	12.438 (0.000)	12.438 (0.000)	12.438 (0.000)	12.438 (0.000)

NHB calculated with a threshold of €50 000 FIB-4= Fibrosis 4 index, ELF=Enhanced Liver Fibrosis, VCTE= Vibration Controlled Transient Elastography, * indicates that liver biopsy was only given to patients with indeterminate VCTE.SP=specificity, SE=sensitivity. **All= all cut-offs were set to correspond to a 90% sensitivity or specificity and corresponding specificity or sensitivity in each test. Number inside parentheses indicate changes in NHB from base case result.

References

*Indicate that the cited reference can be found in the bibliography of the main manuscript

1. Adams LA, Lymp JF, St Sauver J, et al. The natural history of nonalcoholic fatty liver disease: a population-based cohort study. *Gastroenterology*. 2005;129(1):113-121. doi:10.1053/j.gastro.2005.04.014

2. Dulai PS, Singh S, Patel J, et al. Increased risk of mortality by fibrosis stage in nonalcoholic fatty liver disease: Systematic review and meta-analysis. *Hepatology*. 2017;65(5):1557-1565. doi:10.1002/hep.29085

3. Le P, Payne JY, Zhang L, et al. Disease State Transition Probabilities Across the Spectrum of NAFLD: A Systematic Review and Meta-Analysis of Paired Biopsy or Imaging Studies. *Clin Gastroenterol Hepatol*. 2022;S1542-3565(22)00733-9. doi: 10.1016/j.cgh.2022.07.033.

4. Singh S, Allen AM, Wang Z, Prokop LJ, Murad MH, Loomba R. Fibrosis Progression in Nonalcoholic Fatty Liver versus Nonalcoholic Steatohepatitis: A Systematic Review and Meta-analysis of Paired-Biopsy Studies. *Clin Gastroenterol Hepatol*. 2015;13(4):643-654.e9. doi:10.1016/j.cgh.2014.04.014

5. Alexander M, Loomis AK, van der Lei J, et al. Risks and clinical predictors of cirrhosis and hepatocellular carcinoma diagnoses in adults with diagnosed NAFLD: real-world study of 18 million patients in four European cohorts. *BMC Med*. 2019;17(1):95. doi:10.1186/s12916-019-1321-x

6. Younossi ZM, Tampi RP, Nader F, et al. Hypothetical treatment of patients with non-alcoholic steatohepatitis: Potential impact on important clinical outcomes. *Liver Int*. 2020;40(2):308-318. doi:10.1111/liv.14292

7. Villanueva C, Albillos A, Genescà J, et al. β blockers to prevent decompensation of cirrhosis in patients with clinically significant portal hypertension (PREDESCI): a randomised, double-blind, placebo-controlled, multicentre trial. *Lancet*. 2019;393(10181):1597-1608. doi:10.1016/S0140-6736(18)31875-0

8. Nilsson E, Anderson H, Sargenti K, Lindgren S, Prytz H. Incidence, clinical presentation and mortality of liver cirrhosis in Southern Sweden: a 10-year population-based study. *Aliment Pharmacol Ther*. 2016;43(12):1330-1339. doi:10.1111/apt.13635

9. Zipprich A, Garcia-Tsao G, Rogowski S, Fleig WE, Seufferlein T, Dollinger MM. Prognostic indicators of survival in patients with compensated and decompensated cirrhosis. *Liver Int*. 2012;32(9):1407-1414. doi:10.1111/j.1478-3231.2012.02830.x

10. Cabibbo G, Enea M, Attanasio M, Bruix J, Craxì A, Cammà C. A meta-analysis of survival rates of untreated patients in randomized clinical trials of hepatocellular carcinoma. *Hepatology*. 2010;51(4):1274-1283. doi:10.1002/hep.23485

11. Regionalt cancercentrum (Väst*). Primär levercancer- Regional nulägesbeskrivning VGR-standardiserat vårdförlopp*. 2016. Accessed April 01, 2022. https://cancercentrum.se/globalassets/vara-uppdrag/kunskapsstyrning/varje-dag-raknas/vast/nulagesbeskrivning-levercancer.pdf

12. D'Amico G, Pasta L, Morabito A, et al. Competing risks and prognostic stages of cirrhosis: a 25-year inception cohort study of 494 patients. *Aliment Pharmacol Ther*. 2014;39(10):1180-1193. doi:10.1111/apt.12721

13. Swedish Society of Gastroenterology. *Nationellt vårdprogram för Levertransplantation*. 2020 Accessed May 15, 2022. https://svenskgastroenterologi.se/wp-content/uploads/2020/05/2020-vardprogram-levertransplantation.pdf

14. Metodrådet. *Transarteriell kemoembolisering (TACE) vid behandling av primär levercancer (HCC).* Sydöstra sjukvårdsregionen; 2013. Accessed May 25, 2022. https://plus.rjl.se/info_files/infosida40495/tace_2_okt.pdf

15. Lundgren L, Henriksson M, Andersson B, Sandström P. Cost-effectiveness of gallbladder histopathology after cholecystectomy for benign disease. *BJS Open*. 2020;4(6):1125-1136. doi:10.1002/bjs5.50325

16. Regionala expertgruppen för bedömning av cancerläkemedel. *Sorafenib (Nexavar) För Behandling Av Patienter Med Progressiv, Lokalt Avancerad Eller Metastaserad, Differentierad Tyreoideacancer, Refraktär Mot Radioaktivt Jod.* Sydöstra sjukvårdsregionen; 2014. Accessed May 25, 2022. https://cancercentrum.se/globalassets/vara-uppdrag/kunskapsstyrning/cancerlakemedel/sydost/regionala-lakemedelsregimer/nexavar-tyreoideacancer-141209.pdf

17. Regionala expertgruppen för bedömning av cancerläkemedel. *Lenvatinib Vid Första Linjens Behandling Av Icke-Resektabel Hepatocellulär Cancer (HCC)*. Sydöstrasjukvårdsregionen; 2018. Accessed May 25, 2022. https://cancercentrum.se/globalassets/vara-uppdrag/kunskapsstyrning/cancerlakemedel/sydost/regionala-lakemedelsregimer/nexavar-tyreoideacancer-141209.pdf

18. TLV. *Hälsoekonomisk bedömning av Idefirix (imlifidas).* Tandvårds- och läkemedelsförmånsverket; 2019. Dnr 3221/2019.

19. LFN. *Underlag för beslut om subvention - Advagraf.* Läkemedelsförmånsnämnden; 2007. 1093/2007.

20. Sayiner M, Stepanova M, Pham H, Noor B, Walters M, Younossi ZM. Assessment of health utilities and quality of life in patients with non-alcoholic fatty liver disease. *BMJ Open Gastroenterol*. 2016;3(1):e000106. doi:10.1136/bmjgast-2016-000106

21. Teni FS, Gerdtham UG, Leidl R, et al. Inequality and heterogeneity in health-related quality of life: findings based on a large sample of cross-sectional EQ-5D-5L data from the Swedish general population. *Qual Life Res*. 2022;31(3):697-712. doi:10.1007/s11136-021-02982-3

22. Zhang M, Li Y, Fan Z, et al. Assessing health-related quality of life and health utilities in patients with chronic hepatitis B-related diseases in China: a cross-sectional study. *BMJ Open*. 2021;11(9):e047475. doi:10.1136/bmjopen-2020-047475

23. Lim KC, Wang VW, Siddiqui FJ, et al. Cost-effectiveness analysis of liver resection versus transplantation for early hepatocellular carcinoma within the Milan criteria. *Hepatology*. 2015;61(1):227-237. doi:10.1002/hep.27135

24. Cucchetti A, Piscaglia F, Cescon M, et al. Cost-effectiveness of hepatic resection versus percutaneous radiofrequency ablation for early hepatocellular carcinoma. *J Hepatol*. 2013;59(2):300-307. doi:10.1016/j.jhep.2013.04.009

25. Eddy DM, Hollingworth W, Caro JJ, Tsevat J, McDonald KM, Wong JB. Model Transparency and Validation: A Report of the ISPOR-SMDM Modeling Good Research Practices Task Force–7. *Med Decis Making*. 2012;32(5):733-743. doi:10.1177/0272989X12454579

26. Vemer P, Corro Ramos I, van Voorn GAK, Al MJ, Feenstra TL. AdViSHE: A Validation-Assessment Tool of Health-Economic Models for Decision Makers and Model Users. *PharmacoEcon*. 2016;34(4):349-361. doi:10.1007/s40273-015-0327-2

27. Chhatwal J, Dalgic OO, Chen W, et al. Analysis of a Simulation Model to Estimate Long-term Outcomes in Patients with Nonalcoholic Fatty Liver Disease. *JAMA Network Open*. 2022;5(9):e2230426. doi:10.1001/jamanetworkopen.2022.30426

28. Ng CH, Lim WH, Hui Lim GE, et al. Mortality Outcomes by Fibrosis Stage in Nonalcoholic Fatty Liver Disease: A Systematic Review and Meta-analysis. *Clin Gastroenterol Hepatol*. Published online May 2, 2022:S1542-3565(22)00439-6. doi:10.1016/j.cgh.2022.04.014

29. Kip MMA, IJzerman MJ, Henriksson M, et al. Toward Alignment in the Reporting of Economic Evaluations of Diagnostic Tests and Biomarkers: The AGREEDT Checklist. *Med Decis Making*. 2018;38(7):778-788. doi:10.1177/0272989X18797590

30. Finer N. Weight loss interventions and nonalcoholic fatty liver disease: Optimizing liver outcomes. *Diabetes Obes and Metab*. 2022;24(S2):44-54. doi:10.1111/dom.14569
